# Selenium Alleviates Mercury Toxicity in *Ophiocordyceps sinensis*: Mechanistic Insights from Integrated Phenotypic and Transcriptomic Analyses

**DOI:** 10.3390/life16101673

**Published:** 2026-10-07

**Authors:** Li He, Yingqi Hu, Dandan Fu, Jianzhao Qi, Fan Xiao, Aimin Ren, Xinlong Mao, Yanli Huo, Xiangxin Li

**Affiliations:** 1 School of Biological and Pharmaceutical Engineering, Lanzhou Jiaotong University, Lanzhou 730070, China; 15034184916@163.com (Y.H.); fdd02272140@163.com (D.F.); xf20002000@126.com (F.X.); 18626095547@163.com (X.M.); huoylsw@126.com (Y.H.); lixx@mail.lzjtu.cn (X.L.); 2 Center of Edible Fungi, Northwest A&F University, Yangling, Xianyang 712100, China; qjz@nwafu.edu.cn; 3 Institute of Vegetables, Gansu Academy of Agricultural Sciences, Lanzhou 730070, China; gs_ram@126.com

**Keywords:** *Ophiocordyceps sinensis*, mercury stress, sodium selenite, mitigation effect, Hg-Se interaction, antioxidant system, transcriptomics, metabolic regulation

## Abstract

*Ophiocordyceps sinensis*, a valuable medicinal and nutritional resource, faces significant mercury (Hg) contamination risks. Total Hg content in some samples from key producing regions exceeds the 0.2 mg/kg limit set by the Chinese Pharmacopoeia, raising ecological and food safety concerns. To investigate whether and how sodium selenite (Na_2_SeO_3_) alleviates mercury (II) chloride (HgCl_2_) toxicity, this study established various concentrations for Hg, Se, and combined Hg + Se treatments. Results showed that Hg stress significantly inhibited mycelial growth, reducing fresh weight by 38% at 1 mg/L and by up to 98% at high concentrations, whereas Hg + Se treatment increased mycelial dry weight and ameliorated morphological damage relative to Hg treatment alone. Furthermore, Fourier Transform Infrared Spectroscopy (FT-IR), X-ray Diffraction (XRD), and X-ray Photoelectron Spectroscopy (XPS) analyses confirmed the formation of Hg-Se bonds and stable complexes such as HgSe, HgSeO_3_, and HgSeO_4_ in the Hg + Se group, constituting the key material basis for Se-mediated Hg detoxification. Hg stress induced ROS accumulation, with O_2_^−^· peaking at 3.96 nmol/g and GSH at 4.86 μg/g under 2 mg/L Hg; exogenous Se reduced O_2_^−^· accumulation by 37%. Soluble sugar increased continuously with Hg concentration, while soluble proteins increased continuously under Hg + Se treatment. Transcriptome analysis indicated that Se alleviated Hg-induced transcriptomic disturbances by modulating genes associated with tyrosine metabolism (*TYR*), peroxisome function (*CAT*), and glucose metabolism (*tktA*, *rpiA*). In conclusion, Se alleviates Hg toxicity in *O. sinensis* through the synergistic actions of chemical chelation and metabolic regulation, offering new insights into Hg mitigation in artificial cultivation.

## 1. Introduction

*Ophiocordyceps sinensis* is a rare entomopathogenic fungus that forms a symbiotic complex with bat moth larvae. This complex contains various bioactive compounds, including cordycepin and polysaccharides, and exhibits numerous pharmacological effects, such as anti-tumor, anti-fibrosis, and immune-regulatory properties [1]. Consequently, it holds considerable application potential in the domains of medicine and health products [2,3]. *O. sinensis* is primarily found in the alpine meadows at elevations exceeding 3000 m on the Qinghai–Xizang Plateau and the southern slopes of the Himalayas in China [4]. This region, situated in the Asian inland plateau, experiences the “cold-trapping effect,” under which atmospheric mercury (Hg) is more readily deposited through dry and wet deposition under low-temperature conditions and continuously accumulates in environmental matrices such as glaciers, vegetation, and soil at high altitudes, thereby making it a significant deposition sink for global Hg pollution [5,6,7,8]. Regional-scale investigations have shown that surface soil Hg concentrations on the Qinghai–Xizang Plateau vary with vegetation type, with forest soils (53.9 ± 36.9 ng/g) exhibiting higher levels than alpine meadow soils (30.7 ± 14.3 ng/g) and grassland soils (24.5 ± 16.1 ng/g), of which 74–87% of soil Hg originates from long-range atmospheric transport and deposition [9]. This ecological context places wild *O. sinensis* resources at significant risk of Hg stress. Investigations have shown that Hg contamination in *O. sinensis* varies depending on sample sources. Commercially available samples exhibited an Hg exceedance rate of 70% [10]. In contrast, cultivated products showed a detection rate of only 13.3%, and their Hg concentrations ranged from 0.02 to 0.05 mg/kg [11], all below the limit of 0.2 mg/kg for Hg in Cordyceps sinensis as specified in the Chinese Pharmacopoeia (2025 Edition) and the relevant industry standards (WM/T2-2004; T/CATCM 009-2019) [12,13]. Although cultivated products demonstrate certain advantages in terms of Hg safety, their quality and safety still warrant continued attention. The toxicity of the heavy metal Hg has been established in various organisms [14,15], including animals, plants, and microorganisms. Its detrimental effects on fungi primarily involve the inhibition of mycelial growth, disruption of cellular ultrastructure, and interference with fundamental metabolic processes [16,17,18,19,20], which directly jeopardizes the normal development and quality of *O. sinensis*. Nevertheless, systematic research on the specific impacts of Hg stress on the growth and development of *O. sinensis* and the underlying molecular mechanisms remains insufficient.

Numerous studies have demonstrated that selenium (Se) is a pivotal beneficial element for organisms, which effectively enhances biological stress resistance and antagonizes heavy metal toxicity [21,22,23,24,25]. Biologically derived selenium nanoparticles have been proven to possess excellent antioxidant activity and improve organism growth performance, which provides a fundamental physiological basis for stress mitigation [26,27]. As an essential trace element, Se serves as a core component of key selenoproteins such as glutathione peroxidase (GPx) and thioredoxin reductase (TrxR), maintaining intracellular redox homeostasis and executing critical heavy metal detoxification functions [28,29]. In the fungal system, Se mitigates Hg toxicity through a multi-dimensional synergistic network, with mechanisms of action spanning molecular interactions, cellular metabolism, and systemic regulation.

In vivo, sodium selenite (Na_2_SeO_3_) can be reduced by antioxidants including glutathione (GSH) to generate Se^2−^. The generated Se^2−^ then binds to Hg to form poorly soluble, low-toxicity mercury selenide (HgSe) nanoparticles, substantially lowering Hg bioavailability. In this process, GSH acts not only as a reductant for selenite reduction but also directly participates in Hg detoxification via its metabolism: Hg stress induces the up-regulation of the glutathione synthetase gene *GSS*, thereby enhancing GSH synthesis and leading to the formation of Hg-GSH complexes. Subsequently, this complex is sequestered into the vacuole through vacuolar membrane transport proteins, such as Yeast Cadmium Factor 1 (YCF1) homolog, facilitating intracellular detoxification and storage of Hg [30,31]. At the transmembrane transport level, selenite (SeO_3_^2−^) competes with Hg^2+^ for binding sites on absorption channels, including aquaporins, thereby directly inhibiting the transmembrane absorption of Hg. The Selenocysteine-Assisted Organometallic (SAO) system encodes ATP-binding cassette (ABC) transporter subunits carrying selenocysteine and homologs of heavy metal efflux pumps, constituting a complete pathway of substrate capture, import, metabolism, and efflux, suggesting that Se may influence Hg transmembrane efflux through regulation of ABC transporters [32]. Such competition between essential trace elements and toxic heavy metals for uptake channels, together with intracellular sequestration via chelation and vacuolar compartmentalization, as well as efflux mediated by transporters, represents a widely recognized strategy by which organisms limit the accumulation of heavy metals across various biological systems [33,34]. Se also plays a crucial role in the response to oxidative stress by significantly activating the endogenous antioxidant defense system. For example, in *Cordyceps militaris* (a model fungus), the exogenous application of Na_2_SeO_3_ markedly enhances the activities of superoxide dismutase (SOD) and GPx, while concurrently reducing the levels of malondialdehyde (MDA), a lipid peroxidation product. This effect is closely associated with the restoration of Se-dependent antioxidant pathways, such as TrxR, which effectively mitigates the excessive reactive oxygen species (ROS) generated by Hg stress [35]. At the gene regulation level, Se has been reported to affect the expression of DNA methyltransferase (DNMT) genes in various biological systems [36,37]. Whether such epigenetic modulation occurs under Hg stress in *O. sinensis*, however, remains to be investigated. Additionally, Se may indirectly influence the expression network of downstream antioxidant and stress response genes by modulating the expression of specific microRNAs (miRNAs) [38,39]. Although the chemical antagonism between Se and Hg is well established, the biological mechanisms underlying Se-mediated regulation of antioxidant enzyme activities and Hg transporter gene expression in *O. sinensis* against Hg stress are yet to be elucidated.

*O. sinensis* is the dominant fungal species throughout the life cycle of Chinese cordyceps and exhibits species-specific responses to heavy metal stress. Among the genetic factors potentially underlying these responses, heavy metal resistance-related genes are of particular interest. Genomic analyses of *O. sinensis* have revealed that heavy metal resistance-related genes are present at relatively low abundance [40]. This genomic feature may partially contribute to the sensitivity of *O. sinensis* to Hg stress, although heavy metal sensitivity and tolerance are complex traits governed by multiple pathways, including uptake, efflux, chelation, compartmentalization, and antioxidant defense, rather than by the abundance of a few resistance genes alone. Moreover, the molecular weight of approximately 24 kDa for polysaccharides derived from *O. sinensis* mycelium, along with the compositional characteristics of monosaccharides, including the mannose-to-glucose ratio, directly influences the chelating capacity of these polysaccharides with Hg^2+^ via hydroxyl and carboxyl groups. Fourier transform infrared spectroscopy (FT-IR) analysis validated the formation of polysaccharide–Hg complexes [41,42]. The introduction of exogenous Se indirectly facilitates the normal synthesis and secretion of polysaccharides from *O. sinensis* by preserving cellular metabolic homeostasis and energy supply. This process enhances physical detoxification and creates a synergistic protective effect through biochemical regulation and physical chelation. However, a notable imbalance exists in the current research within this field; the majority of mechanistic evidence is derived from model fungi such as *C. militaris*, and the research predominantly centers on the response to arsenic (As) stress [43,44]. To date, systematic and in-depth integrated research on the specific molecular adaptation mechanisms and core regulatory pathways of the precious *O. sinensis* under Hg stress remains insufficient.

This study aims to investigate the protective effects and underlying mechanisms of Na_2_SeO_3_ against mercury(II) chloride (HgCl_2_) stress in *O. sinensis*. Integrating physiological and biochemical measurements, microscopic observation, elemental speciation analysis, and transcriptome sequencing, we systematically characterize fungal responses under control (CK), Hg, Se, and combined Hg–Se treatments. Se may alleviate Hg toxicity primarily by forming insoluble Hg–Se complexes to reduce Hg bioavailability in fungal cells. This work seeks to expand the understanding of Se-mediated Hg detoxification in this fungus and provide insights into Hg–Se antagonism in microbial heavy metal detoxification.

## 2. Materials and Methods

### 2.1. Experimental Materials

The strain used in this experiment was isolated from a single ascospore of wild *O. sinensis* collected from Daiqian Pasture, Tianzhu County, Wuwei City, Gansu Province, China. Genomic DNA was extracted for ITS sequencing. The obtained sequences were searched against the NCBI BLAST web server to confirm species identity. A phylogenetic tree was constructed using MEGA-X software (version 10.2.6). This strain was designated TZ8-1 and preserved under paraffin wax. The phylogenetic tree is shown in Figure S1.

### 2.2. Cultivation of O. sinensis

The preserved test strain TZ8-1 was inoculated onto potato milk solid medium (200 g potatoes were boiled in deionized water and filtered through four layers of gauze to collect the filtrate. The medium contained the potato filtrate, 200 mL milk, 15 g agar, 20 g glucose, 5 g lactalbumin hydrolysate, 0.2 g MgSO_4_·7H_2_O, 0.5 g CaCl_2_, 1 g KH_2_PO_4_, 5 g yeast extract, and 1 tablet of vitamin B complex; the final volume was adjusted to 1000 mL using deionized water) and activated for 2 to 3 successive generations. Stock solutions of HgCl_2_ and Na_2_SeO_3_ were filter-sterilized through 0.22 μm aqueous membrane filters and added to 99 mL of liquid medium (10 g peptone, 20 g glucose, 5 g lactalbumin hydrolysate, 0.2 g MgSO_4_·7H_2_O, 1 g KH_2_PO_4_, 5 g yeast extract, and 1 tablet of vitamin B complex; the volume was adjusted to 1000 mL with deionized water). Sterile syringes were used to spike the medium to reach the target final concentrations. All reported concentrations refer to elemental Hg and elemental Se. The required amounts of HgCl_2_ and Na_2_SeO_3_ were calculated to achieve these target elemental concentrations. For Hg treatment, final concentrations of 0, 0.25, 0.5, 1, 2, 3, 5, 10, 20, 30 and 60 mg/L were established. This concentration gradient was set according to previous studies with minor modifications [45]. The Se treatment was prepared with identical concentrations. Two independent sets of combined Hg + Se treatment experiments were carried out. The first experiment characterized the dose–response relationship of mycelial biomass under combined Hg + Se stress using a fixed Hg:Se elemental mass ratio of 1:1, with total Hg + Se elemental concentrations ranging from 0 to 60 mg/L. The second experiment investigated the effects of different Hg:Se elemental mass ratios, which were set as 1:0, 1:0.5, 1:1, 1:2, and 1:3. For each ratio group, the total elemental mass concentrations of Hg and Se were set at 0.25, 0.5, 1, 1.5 and 2 mg/L. Under each fixed Hg:Se ratio, the Hg and Se concentrations in the medium were simultaneously adjusted to maintain the defined Hg:Se elemental mass ratio [46,47]. An untreated CK was included. Fungal blocks (6 mm in diameter) were punched from the solid medium, and three blocks were placed into each bottle of liquid medium. All cultures were incubated in a constant-temperature shaker at 18 °C under dark conditions with shaking at 100 r/min for 30 days.

### 2.3. Macroscopic Morphological Observation and Mycelial Biomass Determination

Morphological differences in *O. sinensis* mycelial pellets were observed and documented after culture in liquid media under control conditions, different concentrations of HgCl_2_ or Na_2_SeO_3_, and combined stress treatments. The mycelial pellets from each treatment group were transferred into separate centrifuge tubes and then centrifuged at 5000 r/min for 10 min. After removal of the supernatant, the intact *O. sinensis* mycelial pellets were harvested. Surface moisture was blotted from the mycelial pellets with filter paper, and the fresh weight was then recorded. The samples were then freeze-dried, and the dry weight was measured. Fresh and dry weights were measured for three independent biological replicates per treatment. As described in Text S1, melanin brightness was quantified; this parameter is negatively correlated with melanin accumulation.

### 2.4. Characterization of Mycelial Morphology and Elemental Composition of O. sinensis

The mycelial pellets from the liquid medium of each treatment group (CK, Hg, Se, and Hg + Se) were collected by pipette aspiration, centrifuged at 5000 r/min for 10 min, washed three times with deionized water, and stored for subsequent analysis.

To observe the morphological differences of mycelia after different treatments, the pellets were fixed in pre-cooled glutaraldehyde buffer at 4 °C overnight, rinsed with 0.1 mol/L phosphate buffer, dehydrated through a graded ethanol series (30%, 50%, 70%, 90%, 95%) and absolute ethanol, treated with an ethanol–isoamyl acetate mixture, followed by pure isoamyl acetate for 2 h, and freeze-dried for 15 h. The dried samples were mounted on stubs, sputter-coated with gold, and observed and photographed under a scanning electron microscope (SEM, GeminiSEM 500, Carl Zeiss, Oberkochen, Germany). To characterize the distribution of Hg and Se elements on the mycelial surface, energy-dispersive spectroscopy (EDS) analysis was performed simultaneously with SEM at various positions, with an acceleration voltage of 15.0 kV, a take-off angle of 30.0°, and an acquisition time of 25 s. To quantify the Hg and Se contents in the mycelia, 0.5 g of mycelial pellets were weighed and placed into a digestion vessel, and 4 mL of a mixture of concentrated nitric acid and hydrochloric acid (*v*/*v* = 3:1) was added. The vessel was then placed in a microwave digestion system (Multiwave PRO, Anton Paar, Graz, Austria), heated to 160 °C within 5 min, and digested at 160 °C for 20 min. After cooling, the digest was transferred to a graphite digestion apparatus (RNKL-GS, Nanjing Ruinike, Nanjing, China), where the samples were slowly evaporated while retaining a small volume of liquid. After cooling, the residue was diluted to 5 mL and further diluted 1000-fold. The Hg and Se contents were determined using an atomic fluorescence spectrometer (AFS-9700, Beijing Haiguang Instrument, Beijing, China), with a standard curve (0–1 mg/L) for quantification. The elemental contents were calculated as (solution concentration × digestion volume)/sample mass. Hg and Se contents were determined from three independent biological replicates. To analyze the changes in cell wall functional groups after different treatments, the freeze-dried mycelia were mixed with KBr at a ratio of 1:100 and ground to a particle size less than 2 μm, then pressed into pellets. Fourier transform infrared spectroscopy (FT-IR) spectra were recorded on a spectrometer (VERTEX 70, Bruker, Billerica, MA, USA) in transmission mode over the range 400–4000 cm^−1^ at a resolution of 2 cm^−1^. To identify the phase composition and elemental chemical states of the mycelia, the freeze-dried and ground powder (sieved through a 320-mesh sieve) was mixed with an internal standard (e.g., MgO) and pressed into pellets for X-ray diffraction (XRD) analysis on a diffractometer (Bruker D8 ADVANCE, Bruker). Separately, the freeze-dried ground powder was used for X-ray photoelectron spectroscopy (XPS) analysis on a spectrometer (Nexsa, Thermo Fisher Scientific, Waltham, MA, USA).

### 2.5. Determination of the Content of Relevant Substances in O. sinensis Mycelium

The concentration of soluble sugars was determined using the anthrone method [48], while the concentration of soluble proteins was determined via the Coomassie Brilliant Blue G-250 assay [49]. Protein and glucose standard solutions were diluted to concentrations of 0, 20, 40, 60, 80, and 100 μg/mL. The absorbance of the soluble protein standard was measured at 595 nm, and the absorbance of the glucose standard solution, following the addition of anthrone reagent, was recorded at 620 nm. Standard curves were subsequently plotted based on these measurements.

The content or activity of each analyte was determined using corresponding kits from Solarbio. The specific catalog numbers and detection wavelengths are as follows: GSH (BC1175, 412 nm), superoxide anion radical (O_2_^−^·) (BC1415, 530 nm), hydrogen peroxide (H_2_O_2_) (BC3590, 415 nm), CAT (BC0200, 240 nm), and SOD (BC5250, 450 nm). All measurements were performed using a UV-Vis spectrophotometer (UV759, Shanghai Precision & Scientific Instrument Co., Ltd., Shanghai, China), and all operations were carried out in strict accordance with the manufacturer’s instructions. All biochemical assays were performed with three biological replicates per treatment, with each replicate analyzed in technical triplicate.

### 2.6. Transcriptome Sample Preparation, RNA Sequencing and Bioinformatics Analysis

Mycelial samples from the CK, Hg, Se, and Hg + Se treatment groups were collected during the logarithmic phase of fungal growth (OD_600_ = 0.5–0.8). For each biological replicate, 1 mL of the fungal suspension was transferred into an RNase-free screw-cap cryotube and centrifuged at 4 °C, and the supernatant was discarded. This procedure was repeated until a total of 2 mL of fungal suspension had been collected. After the final centrifugation, the supernatant was carefully removed, and the pellet was snap-frozen in liquid nitrogen until completely frozen and stored at −80 °C. Samples were shipped on dry ice, and paired-end RNA-Seq was performed by Shanghai Applied Protein Technology Co., Ltd., Shanghai, China.

Total RNA was extracted from mycelial samples using TRIzol^®^ Reagent [50]. RNA concentration and purity were measured using a NanoDrop 2000 spectrophotometer (NanoDrop 2000, NanoDrop Technologies, Wilmington, DE, USA), and RNA integrity was assessed using an Agilent 5300 Fragment Analyzer (Agilent 5300 Fragment Analyzer, Agilent Technologies, Santa Clara, CA, USA) (RQN > 6.5). mRNA was enriched from total RNA using Oligo(dT)-coated magnetic beads and then fragmented into short fragments (approximately 300 bp) using fragmentation buffer. First-strand cDNA was synthesized using random primers and reverse transcriptase, followed by second-strand cDNA synthesis. After end-repair, A-tailing, adapter ligation, and PCR amplification, the final libraries were constructed and sequenced on an Illumina NovaSeq 6000 platform (NovaSeq 6000, Illumina, San Diego, CA, USA) to generate 150-bp paired-end reads.

Raw sequencing data were quality-controlled using SeqPrep (version 1.3.2, Johns Hopkins University, Baltimore, MD, USA) to remove adapters and low-quality sequences, yielding clean reads. The clean reads were aligned to the *O. sinensis* reference genome (GCA_012934285) using HISAT2 (version 2.2.1, Johns Hopkins University, Baltimore, MD, USA) [51]. Gene expression was quantified using RSEM (version 1.3.3, University of Wisconsin, Madison, WI, USA) [52] to obtain read counts, which were then normalized to FPKM and TPM values. Differential expression analysis was performed using DESeq2 R package (version 1.34.0, Bioconductor) [53] with thresholds of |log_2_FC| ≥ 1 and adjusted *p*-value (padj) < 0.05. All samples underwent RNA extraction, library preparation, and sequencing within a single batch, and no batch effect was present in the dataset. Gene functional annotation was conducted using DIAMOND-blastx (version 2.0.14, Max Planck Institute for Developmental Biology, Tübingen, Germany) [54] (E-value < 1 × 10^−5^) against the NR, Swiss-Prot, KEGG, GO, EggNOG, and Pfam databases. GO enrichment analysis was performed using Goatools (version 0.8.12, GitHub) [55] with Fisher’s exact test, and KEGG pathway enrichment analysis was carried out using R scripts against the KEGG database [56], with padj < 0.05 set as the significance threshold for both analyses. Weighted gene co-expression network analysis (WGCNA) was conducted using the R package WGCNA (version 1.71, University of California, Los Angeles, CA, USA) [57] to identify co-expression modules associated with treatment groups.

### 2.7. RT-qPCR Verification of Differentially Expressed Genes

The 18S rRNA gene of *O. sinensis* was chosen as the reference gene. Total RNA was reverse-transcribed into cDNA using the PrimeScript™ RT Master Mix (TaKaRa, Kusatsu, Shiga, Japan, RR036A) according to the manufacturer’s instructions. The reverse transcription quantitative polymerase chain reaction (RT-qPCR) was performed using the TB Green^®^ Premix Ex Taq™ II (TaKaRa, RR820A). Primers for both the internal reference gene and the target gene were designed using NCBI Primer-BLAST and Roche primer design software (Roche Molecular Systems, Inc., Basel, Switzerland). The RT-qPCR reaction system for validating differentially expressed genes (DEGs) was shown in Table S1, and primer sequences are listed in Table S2. The RT-qPCR procedure included initial denaturation at 95 °C for 5 min, followed by denaturation at 95 °C for 10 s and annealing at 60 °C for 30 s, repeated for 40 cycles. Upon completion of the cycles, a melting curve was generated within the temperature range of 60–97 °C to assess product specificity. RT-qPCR was performed on a LightCycler 480 II (Roche Molecular Systems, Inc., Basel, Switzerland). Each target gene was subjected to three independent biological replicates. The relative expression levels were ultimately calculated using the $2^{-\Delta \Delta C_{t}}$ method [58].

### 2.8. Statistical Analysis

All data were presented as mean ± standard deviation (SD) from three independent biological replicates. Data processing and statistical analyses were performed using SPSS 22.0 (IBM Corp., Armonk, NY, USA) and Excel 2020 (Microsoft Corporation, Redmond, WA, USA). Before one-way analysis of variance (ANOVA), the normality and homogeneity of variances of the data were assessed using the Shapiro–Wilk and Levene’s test, respectively. Differences among the CK, Hg, Se, and Hg + Se groups were analyzed using ANOVA. When ANOVA indicated significant differences (*p* < 0.05), Fisher’s LSD post-hoc test was used for multiple pairwise comparisons. Two-group comparisons (e.g., Hg vs. Hg + Se) were performed using Student’s t-test (two-tailed). The level of statistical significance was set at *p* < 0.05.

## 3. Results

### 3.1. Effects of Different Concentrations of Hg on the Growth of O. sinensis

**Figure 1:**
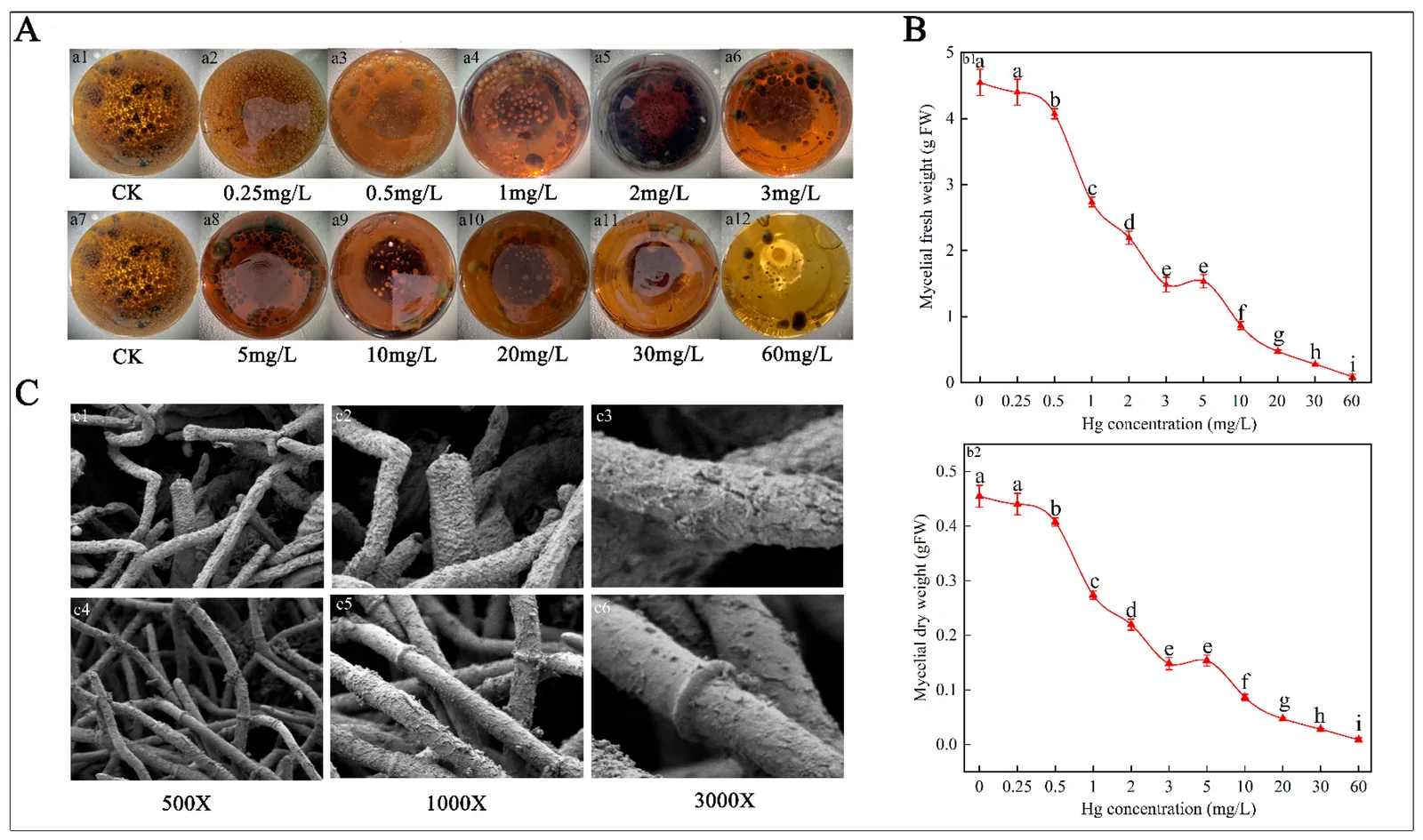
Phenotypes, mycelial fresh and dry weights, and SEM morphology of *O. sinensis* under Hg treatment. (**A**) Colony phenotypes of mycelia exposed to a gradient of Hg concentrations. a1 and a7 correspond to CK; a2–a6 and a8–a12 represent 0.25, 0.5, 1, 2, 3, 5, 10, 20, 30, and 60 mg/L Hg treatments, respectively. (**B**) Mycelial fresh and dry weights across Hg concentration gradients. b1 denotes fresh weight; b2 denotes dry weight. (**C**) SEM micrographs illustrating hyphal ultrastructure under CK and 1 mg/L Hg conditions. c1–c3 correspond to the CK group; c4–c6 correspond to the 1 mg/L Hg treatment group. Different lowercase letters indicate statistically significant differences between groups according to multiple comparisons (*p* < 0.05), while identical letters denote no significant difference.

The growth status of *O. sinensis* mycelium under varying Hg concentrations was observed and documented (Figure 1A). The results indicated that increasing Hg concentration significantly decreased both the fresh and dry weights of mycelial pellets (Figure 1B). Melanin production initially increased and then declined (Figure S2, Text S2), and growth ultimately ceased under high Hg concentrations. The fresh and dry weights of mycelial pellets in the Hg treatment group were reduced by up to 98% compared to the CK group. At the lowest tested concentration of 1 mg/L Hg (Figure 1A a4), the fresh weight of mycelial pellets decreased by 38% relative to CK, while melanin production exhibited a slight increase. Notably, significant morphological differences were observed between the mycelium treated with 1 mg/L Hg and the CK group, directly reflecting the impact of Hg on the growth state of *O. sinensis*. Consequently, the 1 mg/L Hg treatment group was selected for subsequent scanning electron microscopy.

Scanning electron microscopy revealed that the hyphae in the CK group were densely arranged, with a rough and wrinkled surface. The hyphae were thick and exhibited a curved, rod-like structure (Figure 1C c1–c3). Following treatment with 1 mg/L Hg, numerous annular septa formed on the hyphal surface, leading to reduced hyphal volume and causing the hyphae to become intertwined and twisted (Figure 1C c4–c6). Concurrently, the hyphal cell wall thinned, and the surface became smoother. These morphological alterations provide direct evidence that Hg significantly inhibits mycelial growth and disrupts hyphal ultrastructure in *O. sinensis*.

### 3.2. Effects of Different Concentrations of Se and Hg + Se Treatments on the Growth of O. sinensis

**Figure 2:**
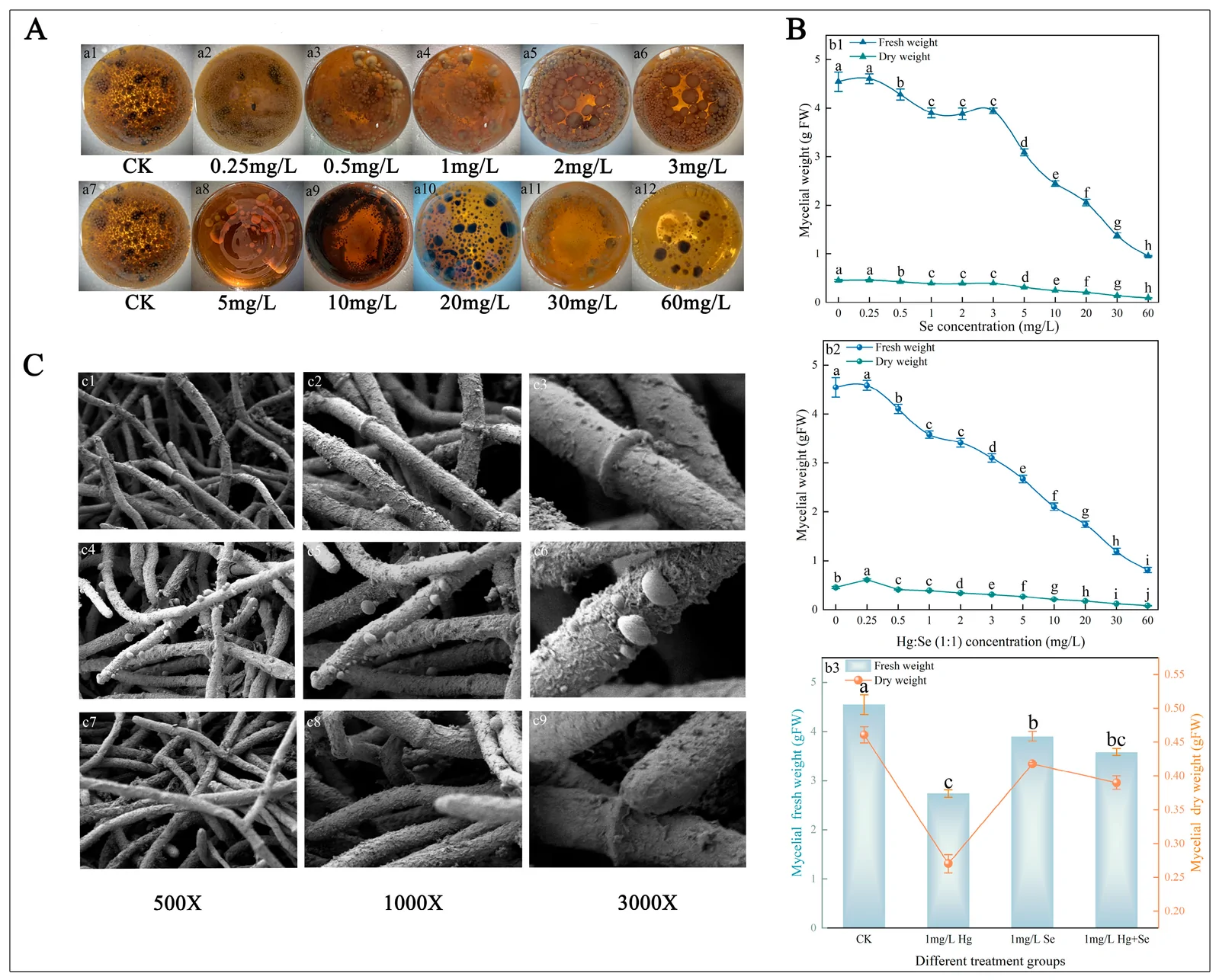
Phenotypes, mycelial fresh and dry weights and SEM morphology of *O. sinensis* under different treatments. (**A**) Colony phenotypes of mycelia under Se treatments at various concentrations. a1 and a7 correspond to CK; a2–a6 and a8–a12 correspond to 0.25, 0.5, 1, 2, 3, 5, 10, 20, 30, and 60 mg/L Se, respectively. (**B**) Mycelial fresh and dry weights under various treatments. b1 represents Se gradient treatment group; b2 represents Hg + Se gradient treatment group; b3 shows the biomass comparison among CK, 1 mg/L Hg, 1 mg/L Se, and 1 mg/L Hg + Se groups. (**C**) SEM micrographs illustrating hyphal morphology under different treatments. c1–c3 correspond to the 1 mg/L Hg group; c4–c6 correspond to the 1 mg/L Se group; c7–c9 correspond to the 1 mg/L Hg + Se group. Different lowercase letters indicate statistically significant differences among treatments (*p* < 0.05), while identical letters denote no significant difference.

Variations in the mycelial growth of *O. sinensis* were recorded under a series of Se treatments (Figure 2A). The results indicated that increasing Se concentration gradually decreased both the fresh and dry weights of mycelial pellets (Figure 2B b1). Notably, at the highest Se concentration tested, the fresh weight of mycelial pellets decreased by 79% compared to the CK group. At 0.25 mg/L Se, the fresh weight of mycelial pellets increased slightly by 1% compared to the CK group, with no significant difference in melanin synthesis between the two groups (Figure S2, Text S2). This slight increase may reflect a weak stimulatory effect of low-concentration Se on mycelial growth, or possibly experimental variation. Even at 60 mg/L Se (Figure 2A a12), the mycelium retained the ability to produce conidia, though conidiation was markedly reduced, without extreme growth inhibition, further confirming that the toxicity threshold of Se stress is significantly higher than that of Hg. Hg + Se combined stress inhibited mycelial growth in a concentration-dependent manner. Compared with the CK group, the maximum concentration of Hg + Se treatment group reduced mycelial fresh weight by 82.14%. A significant decrease in mycelial fresh weight was observed starting at the 0.5 mg/L combined treatment. Both mycelial fresh weight and dry weight showed a continuous and significant downward trend as the Hg + Se treatment group concentration increased (Figure 2B b2).

The mycelial biomass curve under gradient stress illustrated that 1 mg/L was the critical concentration for marked growth alteration. Four treatments (CK, Hg, Se, and Hg + Se) at 1 mg/L were compared. This comparison aimed to distinguish the independent toxicity of Hg, the individual physiological effect of Se, and the antagonistic effect of Se against Hg toxicity, and to characterize the mycelial growth phenotype under Hg + Se treatment. At 1 mg/L, Hg treatment caused severe growth inhibition, Se treatment slightly reduced mycelial biomass relative to the control, yet its inhibitory effect was far milder than Hg stress. while exogenous Se alleviated Hg-induced growth damage. The Hg + Se treatment increased mycelial dry weight compared with Hg treatment, verifying the antagonistic effect of Se against Hg toxicity in fungal mycelia (Figure 2B b3).

Scanning electron microscopy revealed significant differences in mycelial ultramorphology between the 1 mg/L Se treatment group and the 1 mg/L Hg treatment group (Figure 2C c1–c3). In the Se treatment group (Figure 2C c4–c6), the mycelial surface exhibited irregular pustular protrusions and granular structures, without annular septa. The roughness of the mycelium was comparable to that observed in the CK group. To further investigate the mechanism by which Se mitigates Hg toxicity, we established a combined Hg + Se treatment group (Figure 2C c7–c9). Electron microscopy observations indicated that the ultramorphology of the mycelium in the Hg + Se treatment group exhibited characteristics of both the Hg and Se treatments alone. Specifically, a few annular septa remained in the mycelium, suggesting slight interference of Hg with mitosis. The pustular protrusions were absent, and the mycelial surface appeared flatter, indicating that Se ameliorated the morphological damage caused by Hg stress.

### 3.3. Element Content and Accumulation Characteristics of O. sinensis Treated with Hg, Se, and Hg + Se

**Figure 3:**
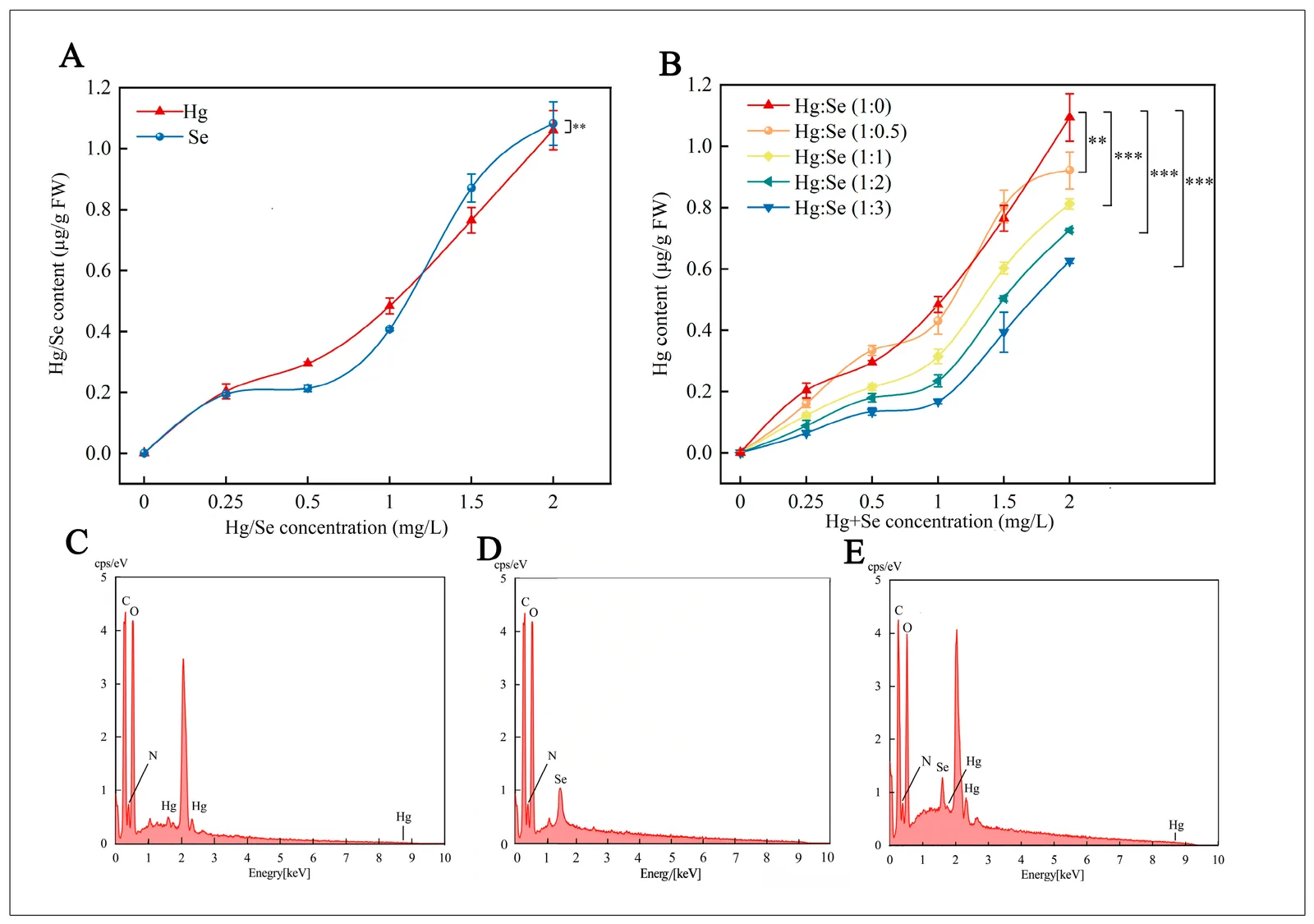
Total Hg and Se contents and elemental accumulation EDS spectra of *O. sinensis* mycelium. (**A**) Changes in Hg and Se Contents under different Hg and Se environmental concentrations. (**B**) Changes in Hg content under different Hg:Se mass ratios. (**C**) EDS spectrum of elemental accumulation on mycelium in the Hg treatment group. (**D**) EDS spectrum of elemental accumulation on mycelium in the Se treatment group. (**E**) EDS spectrum of elemental accumulation on mycelium in the Hg:Se (1:1) treatment group. Asterisks indicate significant differences between groups (* *p* < 0.05, ** *p* < 0.01, *** *p* < 0.001).

AFS was applied to characterize the accumulation patterns of Hg and Se in *O. sinensis* mycelium under 0–2 mg/L concentration gradients for each element (Figure 3A). The results indicated that the concentrations of Hg and Se in the mycelium increased with their respective concentrations in the culture medium, showing a concentration-dependent accumulation pattern. This finding confirms that the absorption capacity of *O. sinensis* for Hg and Se is directly related to the concentrations of these elements in the culture medium.

To investigate the impact of the Hg:Se mass ratio on Hg accumulation in the mycelium, we employed AFS to measure the total Hg content (Figure 3B). Gradient treatment groups were established with Hg:Se mass ratios of 1:0, 1:0.5, 1:1, 1:2, and 1:3. The results indicated that increasing the proportion of Se relative to Hg significantly decreased the Hg content in the mycelium. At a fixed Hg concentration of 2 mg/L in the culture medium, the total Hg content in the mycelium decreased by 2%, 27%, 35%, and 44% in the Hg + Se treatment groups with Hg:Se mass ratios of 1:0.5, 1:1, 1:2, and 1:3, respectively, compared to the Hg treatment group. However, at lower Hg concentrations (0.5 and 1.5 mg/L) and a Hg:Se mass ratio of 1:0.5, the Hg content in the mycelium was slightly higher than that in the corresponding Hg treatment groups. When the Hg:Se mass ratio reached 1:1 (e.g., at 2 mg/L Hg), the Hg content in the mycelium decreased by 25% compared to the 1:0.5 group.

EDS analysis revealed significant differences in elemental distribution on the mycelial surface of *O. sinensis* TZ8-1 after 30 days of treatment with 1 mg/L Hg, 1 mg/L Se, or 1 mg/L Hg + Se. In addition to the basic elements (C, N, and O), distinct Hg enrichment signals were observed on the mycelial surface in the Hg treatment group (Figure 3C), whereas low-intensity Se signals were detected in the Se treatment group (Figure 3D). In the Hg + Se treatment group, both Hg and Se were detected on the mycelial surface, with partial spatial overlap of the two elements (Figure 3E). These findings suggest that Se may alter Hg enrichment characteristics through its colocalization with Hg on the mycelial surface.

### 3.4. Response Characteristics of O. sinensis Under Hg and Se Stresses: Characterized by FT-IR and Jointly by XRD and XPS

**Figure 4:**
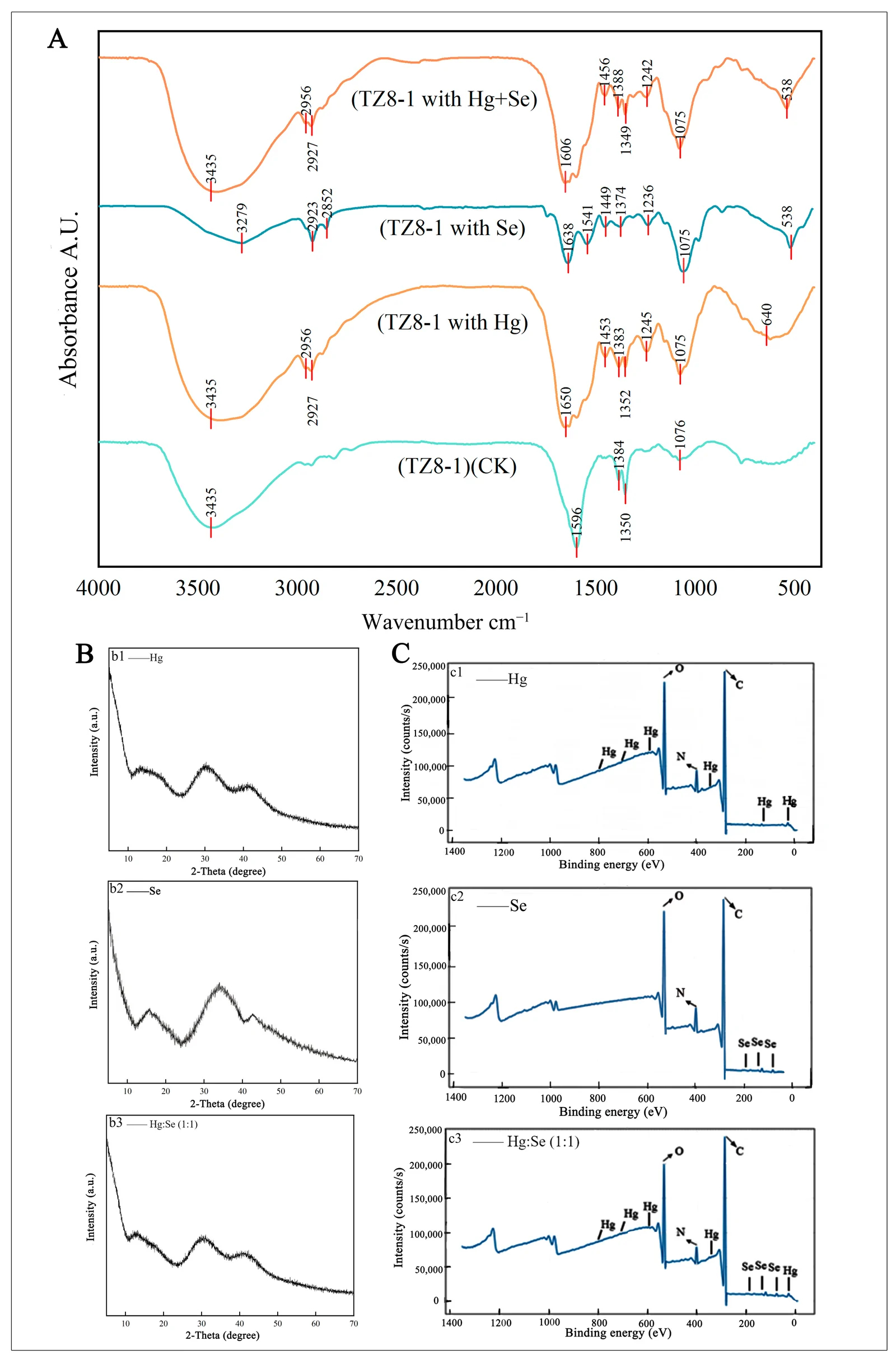
FT-IR, XRD and XPS spectra of *O. sinensis* mycelium under different treatments. (**A**) FT-IR spectra of CK, 1 mg/L Hg, 1 mg/L Se, and 1 mg/L Hg + Se treatment groups over the wavenumber range of 4000–400 cm^−1^. (**B**) XRD spectra of the 1 mg/L Hg (b1), 1 mg/L Se (b2), and 1 mg/L Hg + Se (b3) treatment groups. (**C**) Full-range XPS spectra of the 1 mg/L Hg (c1), 1 mg/L Se (c2), and 1 mg/L Hg + Se (c3) treatment groups.

FT-IR was used to characterize changes in cell wall functional groups of *O. sinensis* under Hg and Hg + Se treatments (Figure 4A). Compared with the CK group, the FT-IR spectra of the 1 mg/L Hg treatment group showed significantly enhanced intensities of characteristic peaks at 2956 cm^−1^, 2927 cm^−1^ (C-H stretching vibration), and 1075 cm^−1^ (C-O stretching of polysaccharides). Notably, the N-H/amide I vibration peak split into two peaks at 1650 cm^−1^ and 1606 cm^−1^, shifting from its original position at 1596 cm^−1^, while the intensity of the peak near 1350 cm^−1^ diminished. For the 1 mg/L Se treatment group, the broad O–H/N–H stretching band exhibited a prominent red shift from 3435 cm^−1^ to 3279 cm^−1^, indicating the formation of stronger hydrogen-bonding interactions between Se species and hydroxyl/amino groups of the mycelium. The main functional group peaks were maintained without obvious peak splitting. The amide-related peak shifted to 1638 cm^−1^, and the peak at 1075 cm^−1^ (polysaccharide C-O) was retained, while the intensity of C-H vibration peaks at 2923 and 2852 cm^−1^ changed slightly relative to CK. In the Hg + Se treatment group, a new characteristic peak corresponding to the Hg-Se bond appeared at 538 cm^−1^. Additionally, the intensity of the C-H-related characteristic peak was lower than that in the Hg treatment group, suggesting that Se may regulate Hg adsorption on the mycelial cell wall by forming Hg-Se complexes or by competing for binding sites.

The XRD results indicated the presence of compounds such as dimethylmercury ((CH_3_)_2_Hg), HgCl_2_, and mercury sulfide (HgS) in the mycelium of the 1 mg/L Hg treatment group. In the 1 mg/L Se treatment group, the presence of compounds such as elemental Se, selenium dioxide (SeO_2_), and selenium trioxide (SeO_3_) was detected. In the 1 mg/L Hg + Se treatment group, Se-containing complexes, including SeO_2_, HgSe, mercury (II) Selenite (HgSeO_3_) and mercury (II) Selenate (HgSeO_4_), were also identified in addition to the Hg compounds mentioned above (Figure 4B). Full-spectrum XPS analysis further confirmed the presence of Se in the Se treatment group and the simultaneous presence of Hg and Se in the mycelium of the Hg + Se treatment group, while only Hg was detected in the Hg treatment group. Moreover, characteristic peak shifts in the XPS energy spectrum confirmed the formation of Hg-Se bonds in the Hg + Se treatment group, whereas only Hg was detected in the Hg treatment group (Figure 4C). The combined XRD and XPS findings suggest that *O. sinensis* can absorb Hg and Se from the culture medium and synthesize stable Hg-Se compounds, representing a key mechanism underlying its tolerance to Hg stress.

### 3.5. Response Characteristics of the Osmotic Regulatory Substances and Antioxidant Defense System of O. sinensis Under Different Hg and Se Stresses

**Figure 5:**
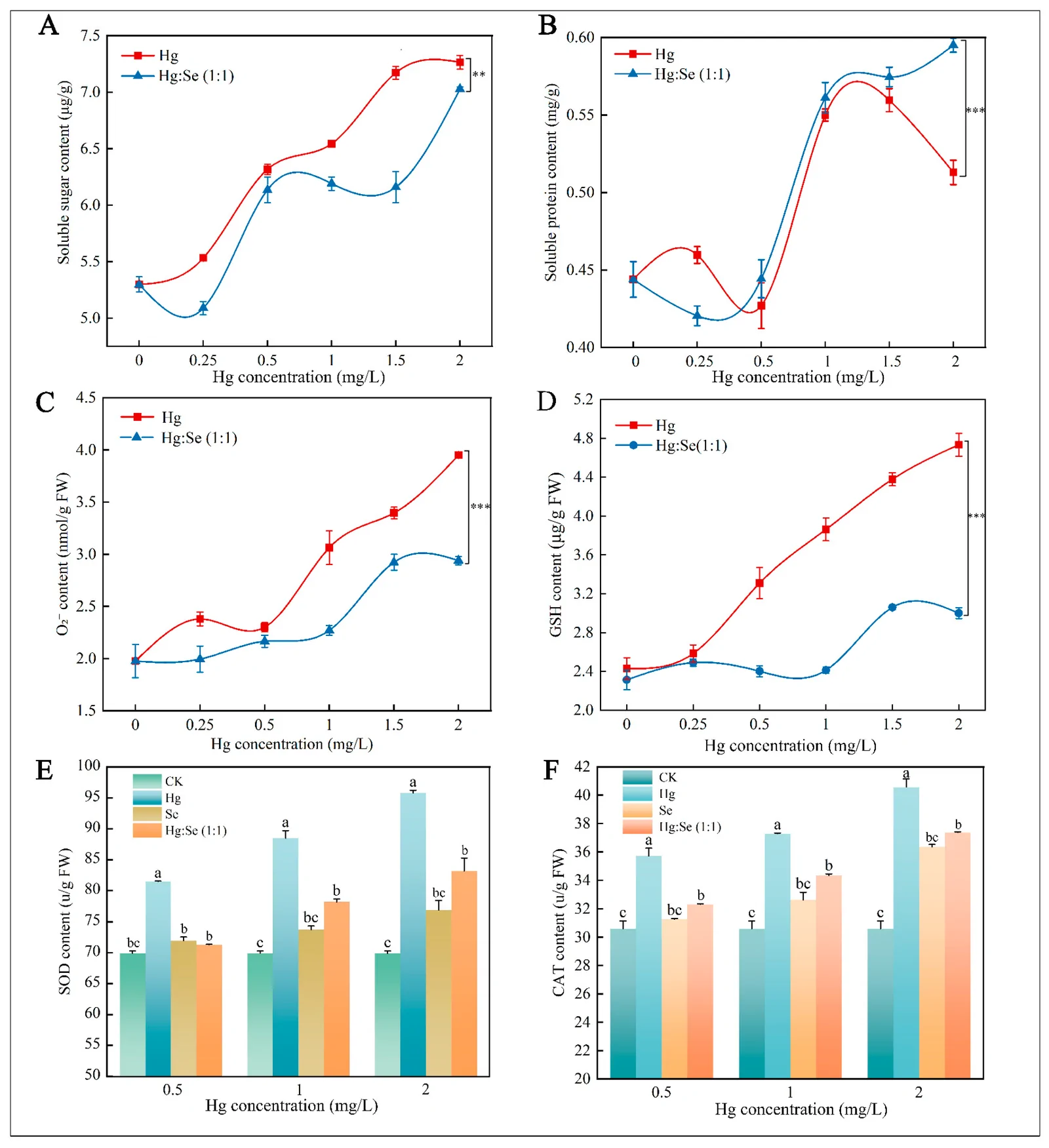
Changes in the content of osmotic regulatory substances and antioxidant related indicators of *O. sinensis* under different treatments. (**A**–**D**) Changes in the contents of soluble sugar, soluble protein, O_2_^−^·, and GSH in response to different concentrations of Hg and Hg + Se (1:1) treatments. Asterisks represent pairwise significance (* *p* < 0.05, ** *p* < 0.01, *** *p* < 0.001). (**E**,**F**) SOD and CAT activities under control, different concentrations of Hg, Se, and Hg + Se treatments. Lowercase letters indicate inter-treatment significant differences at *p* < 0.05, with identical letters meaning no significant difference.

Soluble sugar content in the mycelium consistently increased with rising Hg concentration. In the Hg + Se treatment group, soluble sugar levels generally increased with Hg concentration, except for a phased decrease observed in the 0.25–1.5 mg/L range (Figure 5A). The metabolic response of soluble proteins was more complex: the Hg treatment group exhibited a nonlinear “up–down–up” fluctuation pattern, whereas the Hg + Se treatment group showed a continuous upward trend. At a Hg concentration of 1 mg/L, the soluble protein content in the Hg + Se treatment group increased by 26% compared to the level at 0.5 mg/L Hg, surpassing the 21% increase observed in the Hg treatment group over the same concentration interval. Furthermore, soluble protein content remained elevated at Hg concentrations above 1 mg/L (Figure 5B).

ROS metabolism was notably concentration-dependent under both Hg and Hg + Se stress conditions. Specifically, O_2_^−^· content in the Hg group rose from 2.00 nmol/g to 3.96 nmol/g, peaking at 2 mg/L Hg. In contrast, the Hg + Se group exhibited a significant decrease in O_2_^−^· content, with the peak shifting to 1.5 mg/L Hg. At the peak concentration, the addition of exogenous Se inhibited Hg-induced ROS accumulation by 37% (Figure 5C). H_2_O_2_ content followed a similar pattern to O_2_^−^·: peaking at 1–1.5 mg/L Hg in the Hg group, and at 0.56 mmol/g in the Hg + Se group, with the peak shifting to 1 mg/L Hg. Notably, H_2_O_2_ generation in the Hg group decreased at Hg concentrations exceeding 2 mg/L, while the Hg + Se group maintained a 25% increase over the CK group (Figure S3). These findings highlight the significant inhibitory effect of exogenous Se on intracellular ROS accumulation induced by Hg stress. GSH content exhibited a notable dose-dependent increase with rising Hg levels. At 2 mg/L Hg, GSH levels peaked at 4.86 μg/g, suggesting that Hg stress effectively triggered the antioxidant system of *O. sinensis* fungal cells to combat oxidative stress. Notably, the GSH content in the Hg + Se group was significantly lower than that in the Hg group at the same Hg concentration. Between 0.25 and 1 mg/L Hg, GSH levels remained relatively constant without significant elevation. In the 1.5–2 mg/L Hg range, GSH showed a slight increase but remained significantly lower than in the Hg group, providing further evidence of the mitigating effect of Se on Hg toxicity (Figure 5D).

In the Hg treatment group, SOD and CAT activities progressively increased with rising Hg concentrations. In contrast, the Se treatment group showed a less pronounced increase in enzyme activities at equivalent concentrations, indicating that Hg induced a stronger oxidative stress response in the fungus. The Hg + Se treatment group exhibited significantly less oxidative damage than the Hg group. Although the increase in enzyme activity in this group surpassed that of the Se treatment group, it remained lower than that observed in the Hg group. These findings suggest that exogenous Se partially mitigated Hg toxicity, thereby providing a degree of relief from Hg stress (Figure 5E,F).

### 3.6. Transcriptomic Analysis of O. sinensis Under CK, Hg, Se, and Hg + Se Treatments

In this study, we established four treatment groups for the *O. sinensis*: CK, Hg, Se, and Hg + Se. After 30 days of stress treatment, we collected samples from each group for transcriptome sequencing (three biological replicates per group, totaling 12 samples). Following filtering and quality control of the initial sequencing data, we obtained 75.11 Gb of clean bases. The effective data volume for each sample ranged from 5.90 to 6.77 Gb, with Q30 values ranging from 94.55% to 94.90% and an average GC content of 59.08%. Basic sequencing QC metrics for each sample are summarized (Table S3). All quality control metrics satisfied the analysis requirements, indicating that the transcriptome sequencing data are highly reliable and suitable for subsequent gene expression analysis.

**Figure 6:**
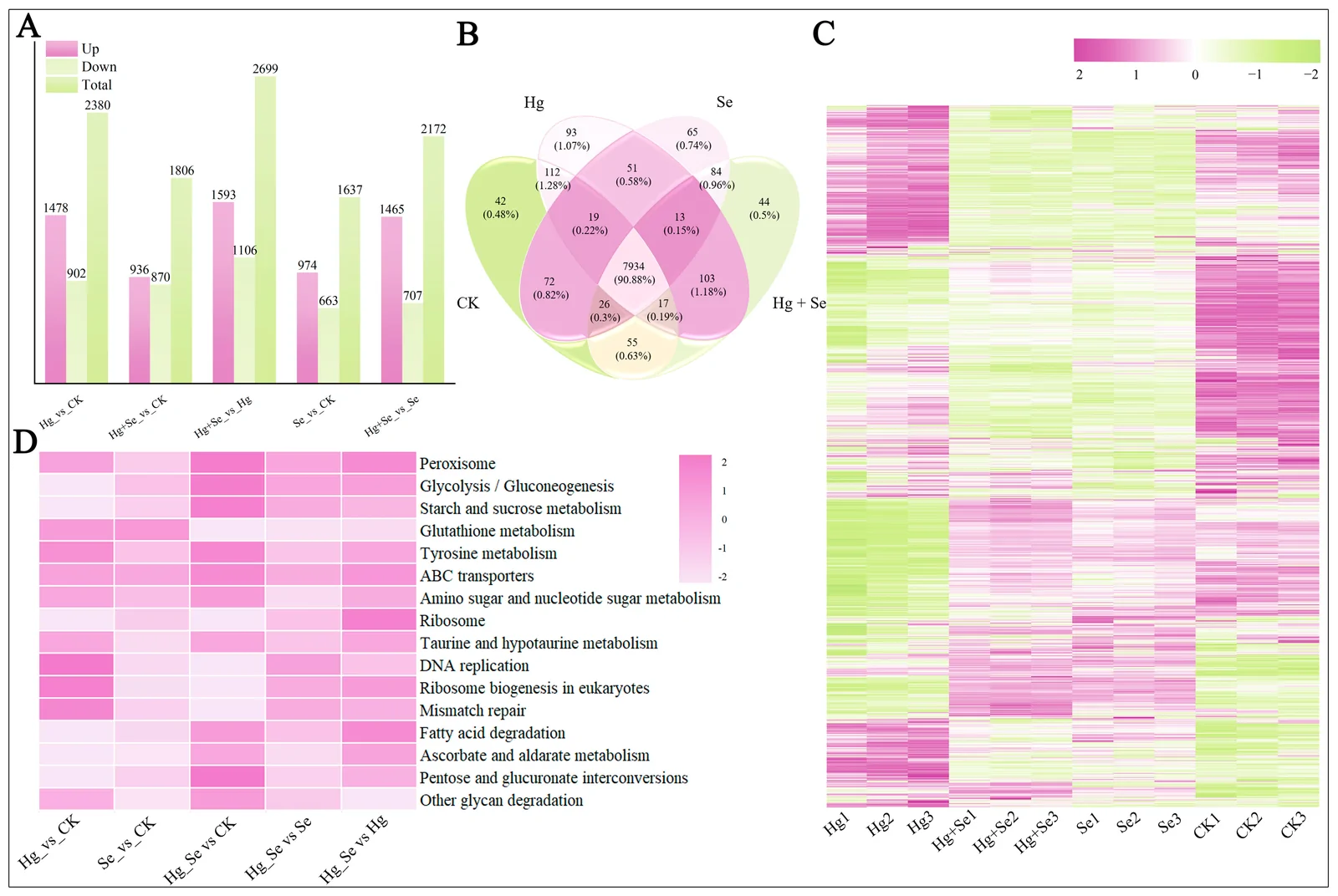
Statistics, clustering and KEGG enrichment analysis of differentially expressed genes in *O. sinensis*. (**A**) The numbers of up- and down-regulated DEGs in comparison groups among the CK, Se, Hg, and Hg + Se treatment groups (Hg vs. CK, Hg + Se vs. CK, Hg + Se vs. Hg, Se vs. CK, Hg + Se vs. Se). (**B**) Venn diagram showing the overlap of DEGs among the comparison groups. (**C**) Clustering heatmap based on expression levels of all DEGs across samples from the four treatment groups (hierarchical clustering with Euclidean distance and complete linkage), the color scale represents the expression level from low to high. (**D**) KEGG enrichment scatter plot of DEGs at adjusted *p* < 0.05 with color gradient indicating enrichment significance.

Pairwise comparative analysis of gene expression across the four treatment groups identified over 1600 DEGs in each of the five comparisons (Figure 6A). Expression analysis of these DEGs indicated that Hg stress induced a robust transcriptional response in *O. sinensis*. In contrast, the addition of exogenous Se enabled the fungus to transition from a state of intense stress expression to a relatively stable adaptive state. This finding provided insights into the molecular mechanism by which *O. sinensis* dynamically regulates its gene expression networks to manage and adapt to heavy metal stress. Venn diagram analysis revealed that shared genes dominated across the four treatment groups, while each group also possessed a certain number of unique genes, indicating that different treatments triggered distinct transcriptional responses alongside the maintenance of a core expression program (Figure 6B). Clustering heatmap analysis showed that the four treatment groups each formed a distinct cluster, with the three biological replicates within each group closely adjacent and clear boundaries between groups. Several gene clusters were highly expressed in the Hg group but lowly expressed in the Hg + Se group, while other gene clusters were highly expressed in the Hg + Se and Se groups but lowly expressed in the Hg group (Figure 6C), indicating that the addition of exogenous Se markedly altered the gene expression pattern under Hg stress.

To elucidate the functional characteristics and metabolic pathways associated with the DEGs, we performed KEGG metabolic pathway enrichment analysis (Figure 6D), identifying a total of 44 distinct KEGG pathways. These pathways comprised 25 metabolic pathways, 2 transport/resistance pathways, 4 regulatory pathways, and 13 biosynthetic pathways. Notably, pathways such as peroxisome, tyrosine metabolism, glycolysis/gluconeogenesis, glutathione metabolism, and ABC transporters were significantly enriched. Collectively, these pathways may mediate the alleviation of Hg toxicity by Se through multiple mechanisms.

### 3.7. Analysis of Core Metabolic Pathways Underlying Se Alleviation of Hg Stress

**Figure 7:**
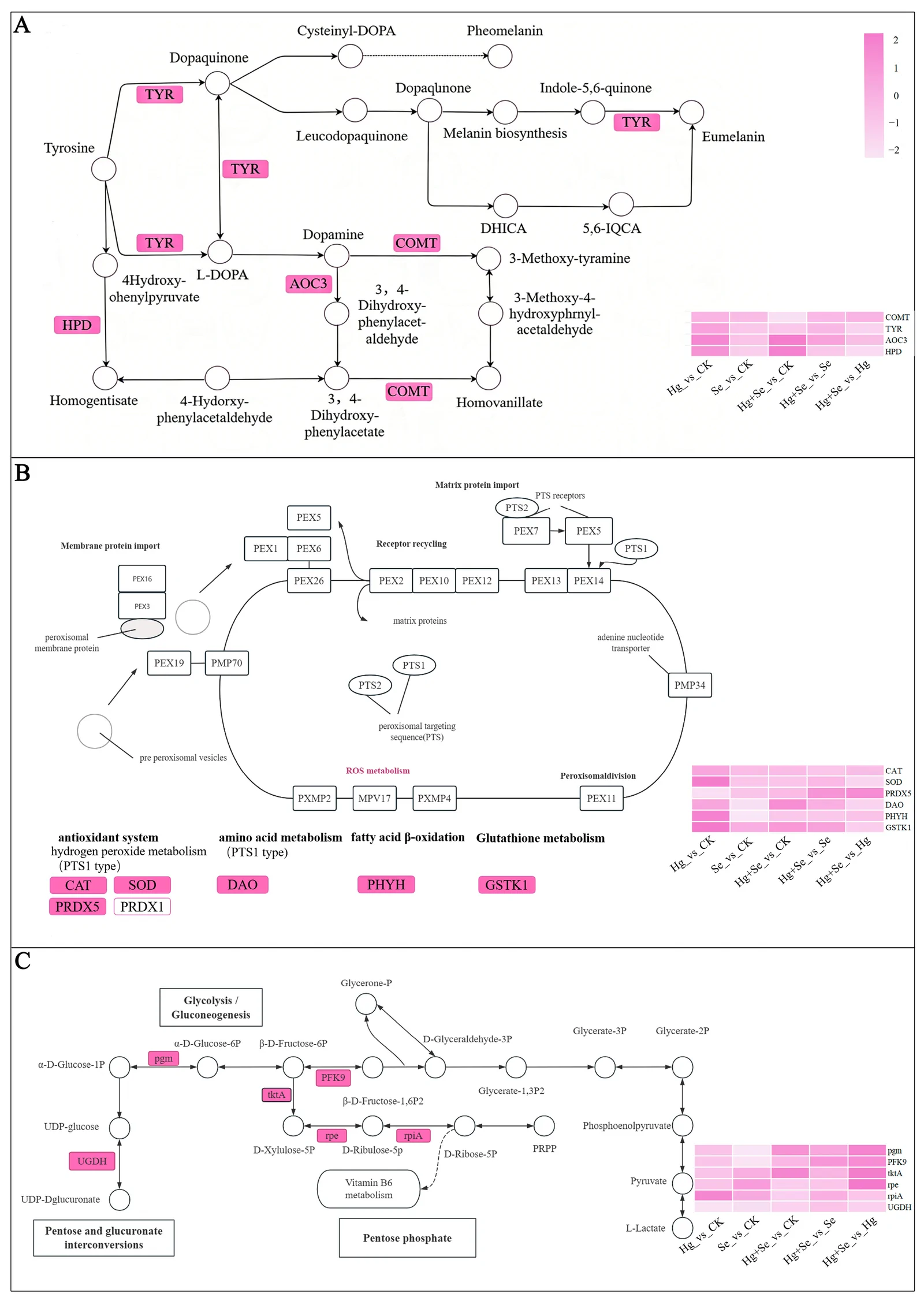
Major metabolic pathways involved in Se-mediated alleviation of Hg stress in *O. sinensis*, based on KEGG enrichment analysis of differentially expressed genes in comparison groups among the CK, Se, Hg, and Hg + Se treatment groups (Hg vs. CK, Hg + Se vs. CK, Hg + Se vs. Hg, Se vs. CK, Hg + Se vs. Se). (**A**) Tyrosine metabolic pathway. (**B**) Peroxisome biogenesis pathway. (**C**) Core integration pathway of glucose metabolism. Pink rounded rectangles represent genes with significantly altered expression under stress. Heatmap shows DEG expression abundance for comparison groups among the CK, Se, Hg, and Hg + Se treatment groups. Darker pink = higher expression/upregulation; lighter pink = lower expression.

Based on the KEGG analysis results, the tyrosine metabolic pathway is a critical pathway through which Se mitigates Hg toxicity. In this pathway, the expression of the Tyrosinase gene (*TYR*) in the Hg group was significantly upregulated compared to the CK group, suggesting that Hg stress induced transcriptional activation of this gene. However, *TYR* expression in the Se group showed no significant change relative to the CK group. In addition, *TYR* expression in the Hg + Se group did not differ significantly from that in either the CK or Se group, but was significantly downregulated relative to the Hg group. Additionally, the gene encoding 4-hydroxyphenylpyruvate dioxygenase (*HPD*), a key enzyme in the tyrosine metabolic pathway, catalyzes the conversion of 4-hydroxyphenylpyruvate to homogentisic acid. *HPD* expression in the Hg group was significantly upregulated compared to the CK group, whereas no significant change was observed in the Se group relative to the CK group. In the Hg + Se group, *HPD* expression was significantly higher than that in the CK group but did not differ significantly from that in the Se group. Relative to the Hg + Se group, HPD expression in the Hg group was significantly downregulated. Compared with the CK group, the expression of the catechol-O-methyltransferase gene (*COMT*) was significantly upregulated in the Hg group, but significantly downregulated in the Hg + Se group. In the Se group, *COMT* expression showed a slight decrease relative to the CK group. Relative to the Hg + Se group, *COMT* was upregulated in both the Hg and Se groups, indicating that its expression is co-regulated by Hg and Se. Compared with the CK group, the expression of the amine oxidase copper containing 3 gene (*AOC3*) was significantly upregulated in both the Hg and Hg + Se groups. In the Se group, *AOC3* expression showed no significant change relative to the CK group. In addition, *AOC3* expression in the Hg + Se group did not differ significantly from that in the Se group, but exhibited bidirectional fluctuations relative to the Hg group. This finding suggests that Hg-induced oxidative stress was not fully mitigated by Se, necessitating sustained *AOC3* expression in the mycelium for redox regulation. The observed fluctuations in expression result from the antagonistic balance between Hg and Se (Figure 7A).

Peroxisome metabolism also participates in the Hg–Se interactive response, as supported by KEGG enrichment analysis. In this pathway, the catalase gene (*CAT*) was upregulated in the Hg group relative to the CK group, whereas no significant change was observed in the Se group. In the Hg + Se group, *CAT* expression did not differ significantly from that in either the CK or Se group, but was downregulated relative to the Hg group. The superoxide dismutase gene (*SOD*) was significantly upregulated in the Hg group compared to the CK group, whereas no significant change was observed in the Se group relative to the CK group. In the Hg + Se group, *SOD* expression did not differ significantly from that in the CK group and was significantly downregulated relative to the Hg group. This finding suggests that Se may alleviate the Hg stress-induced increase in gene expression. The glutathione S-transferase kappa 1 gene (*GSTK1*) was upregulated in the Hg group compared to the CK group, whereas *GSTK1* expression in the Se group showed no significant change relative to the CK group. In the Hg + Se group, *GSTK1* expression remained upregulated relative to the CK group, did not differ significantly from that in the Se group, but was downregulated relative to the Hg group. The peroxiredoxin 5 gene (*PRDX5*) showed no significant change in either the Hg or Se group relative to the CK group. In the Hg + Se group, *PRDX5* expression was significantly upregulated relative to the CK, Se, and Hg groups. The d-amino acid oxidase gene (*DAO*) was upregulated in the Hg group relative to the CK group, whereas *DAO* expression in the Se group showed no significant change relative to the CK group. In the Hg + Se group, *DAO* expression was upregulated relative to both the CK and Se groups, but was downregulated relative to the Hg group. The phytanoyl-CoA 2-hydroxylase gene (*PHYH*) was upregulated in the Hg group relative to the CK group, whereas *PHYH* expression in the Se group showed no significant change relative to the CK group. In the Hg + Se group, *PHYH* expression did not differ significantly from that in either the CK or Se group, but was downregulated relative to the Hg group (Figure 7B).

KEGG enrichment further revealed transcriptional reprogramming within interconnected glucose-related metabolic networks, including glycolysis and the pentose phosphate pathway. In these pathways, the phosphoglucomutase gene (*pgm*) was upregulated in the Hg group relative to the CK group, whereas *pgm* expression in the Se group was downregulated relative to the CK group; in the Hg + Se group, *pgm* expression was upregulated relative to the CK, Se, and Hg groups. The phosphofructokinase gene (*PFK9*) was upregulated in the Hg group relative to the CK group, whereas *PFK9* expression in the Se group was downregulated relative to the CK group; in the Hg + Se group, *PFK9* expression was upregulated relative to the CK, Se, and Hg groups. The transketolase gene (*tktA*) showed no significant change in the Hg group relative to the CK group, whereas *tktA* expression in the Se group was upregulated relative to the CK group; in the Hg + Se group, *tktA* expression was significantly upregulated relative to the CK and Hg groups, but downregulated relative to the Se group. The ribulose-5-phosphate-3-epimerase gene (*rpe*) showed no significant change in the Hg group relative to the CK group, whereas *rpe* expression in the Se group was significantly upregulated relative to the CK group; in the Hg + Se group, *rpe* expression was significantly downregulated relative to the CK, Se, and Hg groups. The ribose-5-phosphate isomerase A gene (*rpiA*) was upregulated in both the Hg and Se groups relative to the CK group; in the Hg + Se group, *rpiA* expression was significantly downregulated relative to the CK and Hg groups, but did not differ significantly from that in the Se group. These responses of pentose phosphate pathway-related genes suggest that Se may promote NADPH and pentose synthesis by enhancing this pathway. The UDP-glucose 6-dehydrogenase gene (*UGDH*) showed no significant change in either the Hg or Se group relative to the CK group; in the Hg + Se group, *UGDH* expression was upregulated relative to the CK, Se, and Hg groups. In summary, Se alleviates Hg stress by precisely regulating glucose metabolic flux and carbon flow (Figure 7C).

### 3.8. Reverse Transcription Quantitative Polymerase Chain Reaction (RT-qPCR) Verification of Differentially Expressed Genes

To assess the reliability of the differential gene expression results obtained through RNA sequencing (RNA-seq), this study employed 18S rRNA as the internal reference gene. Six genes were selected from the four treatment groups (CK, Se, Hg, and Hg + Se) for RT-qPCR verification and analysis. The selected genes included the EMG1 N1-specific pseudouridine methyltransferase gene (*EMG1*), the fructose diphosphate aldolase gene (*FBP*), the 2-deoxy-D-gluconate 3-dehydrogenase gene (*kduD*), the tyrosine-tRNA synthase gene (*TYRS*), the N-acetyltransferase complex ARD1 subunit gene (*ARD1*), and the glucan endo-1,3-beta-glucosidase gene (*EGLC*) (Figure S4A). The fold change (FC) and log_2_FC values of the selected differentially expressed genes are summarized in (Table S4). Correlation analysis was performed between the RT-qPCR verification results and the RNA-seq sequencing data. The fitting curve equation was determined to be Y = 0.5403X + 0.8912, with a correlation coefficient of R^2^ = 0.9025 (*p* < 0.01). This indicates a strong correlation between the RT-qPCR results and the RNA-seq data, thereby confirming the validity and reliability of the differentially expressed genes identified through transcriptome sequencing (Figure S4B).

## 4. Discussion

### 4.1. Enrichment Characteristics and Dose Effects of Ophiocordyceps sinensis Toward Hg and Se

It should be noted that the highest soil Hg concentration applied in this study (60 mg/kg) far exceeds the background level in Qinghai–Xizang Plateau soils, which has a mean range of 19.8–37.0 ng/g [59,60]. This concentration was selected because this is a mechanistic toxicological study requiring a wide concentration gradient to establish dose–effect relationships, rather than a simulation of field exposure. Extrapolation to natural environments should therefore consider Hg bioavailability, exposure duration, and environmental matrix differences.

*O. sinensis* exhibits distinct Hg-Se interactions during the processes of enrichment and detoxification. The accumulation of total Hg and total Se in the mycelium is dose-dependent, increasing progressively with rising environmental concentrations of Hg or Se. Moreover, Se may reduce Hg bioavailability via competitive inhibition of cellular uptake. At constant Hg levels, increasing the Se proportion (Hg:Se mass ratio from 1:0 to 1:3) significantly reduces the total Hg content in *O. sinensis* mycelia. This suggests that Se inhibits Hg uptake by altering membrane transport processes or competing with Hg for binding sites on the mycelial surface. FT-IR analysis revealed that Hg stress increased the abundance of hydroxyl and carboxyl groups on the mycelial surface. EDS further detected Hg-Se colocalization signals on the mycelial surface, confirming that *O. sinensis* immobilized heavy metals through a combination of physical adsorption and chemical chelation. Combined XRD and XPS analyses identified HgSe, HgSeO_3_, and other Hg-Se compounds both intracellularly and extracellularly. These findings demonstrated that Se converted highly toxic Hg^2+^ into low-solubility Hg-Se nanoparticles or stable Hg-containing complexes, thereby alleviating the toxicity of free Hg^2+^ in the culture system. This mechanism is consistent with the typical Hg-Se antagonistic effect reported in *Allium fistulosum* and *Allium sativum* [61,62]. It should be noted that the Hg–Se complexes detected in the present study may form via both chemical and potential biological pathways. On the one hand, spontaneous chemical reactions occur between SeO_3_^2−^ and Hg^2+^ in the medium to generate Hg–Se precipitates. On the other hand, the biological absorption of Hg and Se by *O. sinensis* mycelia, along with intracellular immobilization and metabolic transformation of the two elements, might potentially participate in Hg–Se complex formation. The expression levels of the ABC transporter genes *ABCC1* and *ABCG2* were markedly upregulated under combined Hg + Se treatment compared with Hg treatment alone (Figure S5). This result is consistent with previous studies showing that Se promotes the upregulation of heavy metal transporter genes under heavy metal stress [63]. Accordingly, this study offers direct experimental evidence for the molecular mechanisms by which Se alleviates Hg toxicity.

### 4.2. Regulatory Relationship Between Melanin Accumulation in O. sinensis Under Stress and the Tyrosine Metabolic Pathway

The responses of *O. sinensis* to Hg and Se stress showed obvious differences and interactive effects, which are closely associated with the regulation of the tyrosine metabolic pathway. The tyrosine metabolic pathway is the principal route for fungal pigment synthesis, producing compounds such as eumelanin and pheomelanin. This pathway enhances the antioxidant and stress resistance capabilities of the fungus, thereby regulating its survival and adaptability to environmental challenges [64]. As a critical resistance metabolite in *O. sinensis*, melanin acts as a negative regulator of the tyrosine metabolic pathway. Experimental data indicated that melanin content rose to a peak and subsequently declined with increasing Hg concentration. By contrast, melanin content showed a continuous increasing trend as environmental Se levels rose. Low and moderate Hg concentrations activated the tyrosine metabolic pathway, significantly upregulating TYR transcription, enhancing tyrosinase activity, and accelerating the oxidative polymerization of tyrosine, thereby promoting melanin biosynthesis. The synthesized melanin chelated intracellular free Hg^2+^ through its abundant active groups, including phenolic hydroxyl and amino groups. This process represented a key self-defense mechanism of *O. sinensis* against Hg toxicity [65]. This process reduced Hg toxicity in mycelia and served as an acute defensive response of the fungus. Nevertheless, high-dose Hg treatment markedly suppressed the expression of key genes including *TYR* and *HPD*, distorted tyrosinase conformation, abolished its catalytic activity, and disrupted the *HPD*-controlled metabolic cascade. Such damage impairs both the structure and function of the melanin synthesis system. Suppressed *TYR* expression blocked the conversion of tyrosine to dopa and dopaquinone, thereby inhibiting melanin production and resulting in a medium color similar to that of the control group. Dysregulated *HPD* expression led to the accumulation of tyrosine metabolic intermediates, further disrupting the pathway and interfering with normal fungal metabolism. Meanwhile, Hg stress significantly upregulated *COMT* and *AOC3*, which are involved in maintaining intracellular redox homeostasis and regulating secondary metabolism. Elevated expression of these genes represented a compensatory response of *O. sinensis* to Hg-induced oxidative stress. This finding is consistent with previous reports that Hg stress reduces pigment synthesis efficiency in *Elodea canadensis* [66,67], suggesting that melanin synthesis via the tyrosine metabolic pathway is a conserved defense strategy against Hg stress in fungi, whereas *O. sinensis* exhibits species-specific regulatory patterns. Furthermore, Hg disrupts the expression profiles of essential genes of the carbohydrate metabolic pathway, impairing energy supply and cellular structure synthesis. This indirectly exacerbates dysregulation of the tyrosine metabolic pathway and additionally inhibits mycelial growth. Under combined Hg + Se stress, Se partially restores tyrosine metabolic homeostasis and improves fungal stress resistance by modulating the expression of key genes such as *TYR* and *HPD*. This finding is consistent with existing literature [68,69].

### 4.3. Regulatory Relationship Between Antioxidant Systems and the Peroxisome Metabolic Pathway in Stressed O. sinensis

Dynamic regulation of the antioxidant network is the primary physiological mechanism by which *O. sinensis* responds to Hg and Hg + Se stress. Heavy metal stress induces ROS overaccumulation, triggering the activation of multiple cellular antioxidant defense components including GSH [70,71]. Peroxisomes are essential organelles involved in ROS scavenging and cellular redox homeostasis under Hg stress, and they also participate in Se-mediated Hg detoxification upon combined Hg–Se exposure [72]. Experimental results showed that O_2_^−^· levels in the Hg group displayed a fluctuating upward trend with a rise–fall–rise pattern. The fungus achieved periodic ROS scavenging by regulating the transcription of the peroxisomal antioxidant genes *SOD* and *CAT* and the enzyme activities of their encoded proteins. The proteins encoded by *SOD* and *CAT* genes are essential enzymes in the antioxidant defense system, collaborating to maintain intracellular free radical homeostasis [73], a pattern that is conserved across biological systems [74,75,76,77]. Proteomic analysis demonstrated that differentially expressed proteins of *O. sinensis* during fruiting body development are significantly enriched in the peroxisome pathway, and that SOD and CAT activities showed periodic fluctuations, further verifying the essential role of this pathway in dynamic ROS homeostasis [78]. Notably, unlike *C. militaris*, which relies on selenoproteins for direct detoxification, *O. sinensis* lacks the selenocysteine insertion sequence. Accordingly, its Hg-Se antagonism is mainly mediated by indirect metabolic detoxification pathways. Selenopeptides from *C. militaris* exert antioxidant effects by activating the SKN-1/Nrf oxidative stress pathway, a process that depends on the direct catalytic functions of selenoproteins [79]. In contrast, *O. sinensis* under Hg + Se stress in the present study did not exhibit such prominent enzyme activation, highlighting fundamental interspecific differences in Se utilization mechanisms. In the Hg + Se treatment group, mycelial O_2_^−^· levels showed a distinct rise–fall pattern. The activation level of antioxidant enzymes was markedly lower than that in the Hg group but does not return to control or single-Se levels. This phenomenon indicated that the metabolic repair effect of Se has an apparent threshold. At the transcriptional level, *SOD* and *GSTK1* showed no significant change in the Se group relative to the CK group. Under Hg stress, both genes were significantly upregulated, whereas their expression decreased markedly and returned to near-normal levels after Se addition, indicating that Se attenuates the Hg-induced excessive activation of antioxidant genes. *CAT* showed bidirectional expression fluctuations under both stresses, reflecting dual antagonistic transcriptional regulation by Hg and Se. *PRDX5* expression changes only slightly under Hg stress and remains at basal levels under Hg + Se treatment, which is beneficial for maintaining a stable intracellular redox environment. In addition, the peroxisomal functional genes *DAO* and *PHYH*, which are upregulated by Hg, are downregulated following Se intervention, reducing excessive intracellular H_2_O_2_ accumulation and abnormal buildup of metabolic byproducts. Furthermore, Se forms stable Hg-Se complexes with Hg, alleviating the inhibitory effects of Hg^2+^ on core metabolic enzymes and maintaining the normal metabolic capacity of the fungus.

### 4.4. Regulatory Relationship Between Metabolites and the Carbohydrate Metabolic Pathway in Stressed O. sinensis

Soluble sugars serve as the primary energy source for cellular metabolism and participate in the regulation of intracellular osmotic pressure, thereby mediating stress responses [80]. Hg stress disrupts the mitochondrial respiratory chain and impairs oxidative phosphorylation, leading to intracellular ATP depletion and triggering compensatory activation of carbohydrate metabolism [81]. The physiological and metabolic responses of *O. sinensis* to Hg and Hg + Se stress are driven by dynamic reprogramming of the carbohydrate metabolic pathway. Soluble sugar content in *O. sinensis* increased continuously with rising Hg concentrations. In the Hg + Se group, soluble sugar levels showed an initial decrease followed by an increase, and are significantly lower than those in the Hg group at medium and high concentrations. These results suggest that Se alleviates Hg-induced metabolic disturbances by remodeling the carbohydrate metabolic network. Hg stress inhibits the mitochondrial respiratory chain and impairs oxidative phosphorylation-dependent energy supply, prompting *O. sinensis* to activate the carbohydrate metabolic pathway as a compensatory mechanism for maintaining basic physiological activities. Integrated transcriptomic and metabolomic analyses showed that carbohydrate metabolism-related genes are significantly upregulated during primordium germination, confirming the central role of carbohydrate metabolism and glycolysis in energy supply for *O. sinensis* [82]. Glycolysis generates energy rapidly via substrate-level phosphorylation, with upstream substrates derived primarily from the mobilization and degradation of intracellular soluble sugars. Glycolytic intermediates enter the pentose phosphate pathway to produce NADPH, which is critical for maintaining intracellular redox balance and supporting the antioxidant system. This mechanism explains both the massive accumulation of soluble sugars under Hg stress and the activation of stress-resistant metabolism. Previous studies have shown that Se in *C. militaris* markedly upregulates glycolytic and pentose phosphate pathway genes, accelerates glucose uptake and metabolic reprogramming, increases NADPH levels, and reduces excessive carbohydrate accumulation [83]. This mechanism reasonably accounts for both the decreased soluble sugar content in *O. sinensis* after Se addition and the upregulation of the pentose phosphate pathway genes *tktA* and *rpe* in the present study. In this process, multiple key genes coordinately regulate carbohydrate metabolic flux and carbon partitioning. Although *PFK9* was not significantly activated under Hg stress, Se markedly upregulated its expression under combined stress to accelerate glycolysis and ensure energy supply. In addition, *pgm* was significantly upregulated in the Hg + Se group, further improving the turnover rate of carbohydrate substrates. Moreover, Se specifically induced *tktA* and *rpe*, enhancing the pentose phosphate pathway and NADPH production to strengthen antioxidant capacity. Coordinated regulation of these key genes enables the strain to cope with combined stress without excessive soluble sugar accumulation, thereby avoiding excessive carbohydrate accumulation.

## 5. Conclusions

Research indicates that Hg stress markedly inhibits the growth and development of *O. sinensis*, while Se effectively alleviates Hg-induced physiological toxicity. Mycelial uptake of Hg and Se is dose-dependent. Following Hg and Hg + Se treatments, mycelial surface functional groups are altered, with Hg and Se detected both on and inside mycelia. Intracellular Hg–Se complexes may form, supporting Se-mediated Hg detoxification. Under Hg stress, *O. sinensis* rapidly activates defense responses. Over the tested Hg range (0–2 mg/L), antioxidant molecule contents (GSH, H_2_O_2_, O_2_^−^) and antioxidant enzyme activities (CAT, SOD) generally rose with Hg concentration, whereas osmotic adjustment substances (soluble proteins and sugars) first increased then decreased. These patterns differed greatly under Hg + Se treatment, confirming Se’s alleviating effect. Transcriptomic analysis revealed Hg-activated pathways: tyrosine metabolism, peroxisome, glycolysis/gluconeogenesis, and pentose and glucuronate interconversions. Se alone induced minor transcriptional changes, while Hg + Se treatment largely reversed Hg-triggered transcriptomic alterations. Some genes exhibited unique expression profiles in the Hg + Se group, showing responses were governed by Hg–Se antagonism rather than Se alone. The fungus builds a multi-pathway stress network by regulating genes and metabolites. Se sustains intracellular redox and metabolic homeostasis by modulating key genes, coordinating metabolite detoxification and forming low-toxicity Hg–Se complexes. This study elucidates the physiological and transcriptomic mechanisms underlying Se-mediated Hg detoxification in *O. sinensis*, offering theoretical support for optimizing Se supplementation during artificial cultivation. Future work will validate these findings at the fruiting body stage and functionally characterize key genes.

## Data Availability

The raw sequence data reported in this paper have been deposited in the Genome Sequence Archive (Genomics, Proteomics & Bioinformatics 2025) in National Genomics Data Center (Nucleic Acids Res 2025), China National Center for Bioinformation/Beijing Institute of Genomics, Chinese Academy of Sciences (GSA: CRA050190) that are publicly accessible at https://ngdc.cncb.ac.cn/gsa, accessed on 18 September 2026.

## Supplementary Materials

The following supporting information can be downloaded at: https://www.mdpi.com/article/10.3390/life16101673/s1, Figure S1: NJ phylogenetic tree based on ITS sequence of monospora strains; Text S1: Melanin brightness measurement; Table S1: RT-qPCR reaction system for validation of differentially expressed genes; Table S2: Primer of objective gene and internal reference genes; Text S2: Effects of different concentrations of Hg and Se on pigment production in *O. sinensis*; Figure S2: Changes in melanin brightness of *O. sinensis* under different treatments; Figure S3: Changes in H_2_O_2_ content in *O. sinensis* under Hg and Hg + Se treatments; Table S3: Preprocessing results of sequencing data quality; Table S4: Fold change and log_2_ (fold change) for DEGs in Hg vs. CK, Hg + Se vs. CK, Hg + Se vs. Hg, Se vs. CK, and Hg + Se vs. Se comparisons; Figure S4: Gene expression levels of *O. sinensis* by RT-qPCR and linear correlation between RNA-seq and RT-qPCR detection results; Figure S5: Analysis of the ABC transporter metabolic pathway in *O. sinensis* under Hg + Se treatment compared to Hg treatment alone.

## Abbreviations

The following abbreviations are used in this manuscript:

Hg	Mercury
Na_2_SeO_3_	Sodium selenite
HgCl_2_	Mercury (II) chloride
Se	Selenium
ROS	Reactive oxygen species
SOD	Superoxide dismutase
CAT	Catalase
GSH	Glutathione
HgSe	Mercury selenide
YCF1	Yeast Cadmium Factor 1
ABC	ATP-binding cassette
FT-IR	Fourier Transform Infrared Spectroscopy
CK	Control group
SEM	Scanning Electron Microscope
EDS	Energy-Dispersive Spectroscopy
AFS	Atomic Fluorescence Spectrometer
XRD	X-ray Diffraction
XPS	X-ray Photoelectron Spectroscopy
O_2_^−^·	Superoxide anion radical
H_2_O_2_	Hydrogen peroxide
HgSeO_4_	Mercury (II) Selenate
DEGs	Differentially expressed genes
*TYR*	Tyrosinase
*HPD*	4-hydroxyphenylpyruvate dioxygenase
*COMT*	Catechol-O-methyltransferase
*AOC3*	Amine oxidase copper containing 3
*GSTK1*	Glutathione S-transferase kappa 1
*PRDX5*	Peroxiredoxin 5
*DAO*	D-amino acid oxidase
*PHYH*	Phytanoyl-CoA 2-hydroxylase
*pgm*	Phosphoglucomutase
*PFK9*	Phosphofructokinase
*tktA*	Transketolase
*rpe*	Ribulose-5-phosphate-3-epimerase
*rpiA*	Ribose-5-phosphate isomerase A
*UGDH*	UDP-glucose dehydrogenase
RNA-seq	RNA sequencing
RT-qPCR	Reverse transcription quantitative polymerase chain reaction
*O. sinensis*	*Ophiocordyceps sinensis*
