# Supplementary material for: Selenium Alleviates Mercury Toxicity in Ophiocordyceps sinensis: Mechanistic Insights from Integrated Phenotypic and Transcriptomic Analyses
Source: Life (Basel). 2026 Oct 7;16(10):1673. doi: 10.3390/life16101673 (PMC13705842; doi:10.3390/life16101673)
Supplement: Supplementary file 1 [file life-16-01673-s001.zip › life-4605740-supplementary.pdf]

Figure S1

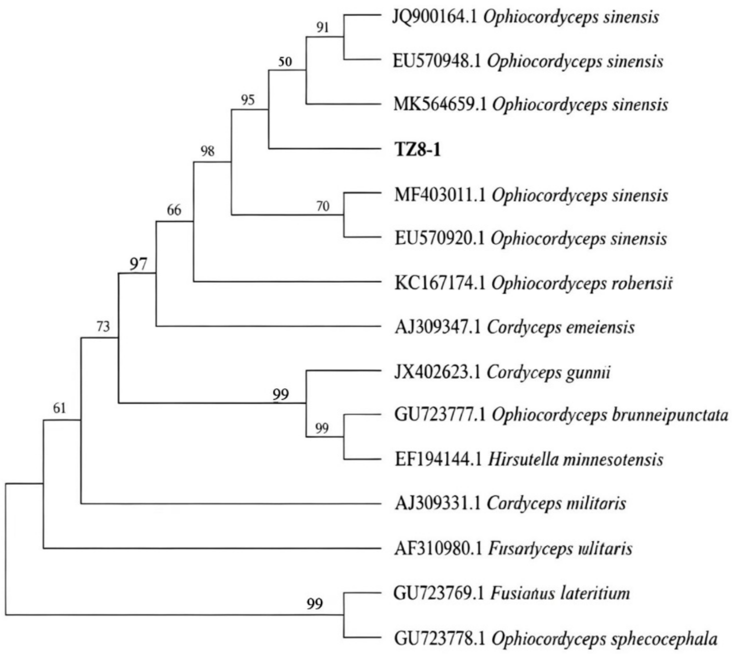

Figure S1. NJ phylogenetic tree based on ITS sequence of monospora strains.

Text S1. Melanin brightness measurement

A 5 mL aliquot of the liquid culture medium, which had been incubated at 18 °C in the dark for 30 days, was centrifuged at 10,000 r/min and 4 °C. The supernatant was collected and filtered through a 0.22 μm aqueous membrane filter into a 2 mL centrifuge tube. Photographs of the centrifuge tubes containing the 2 mL of filtered liquid were taken. The images were then cropped to a uniform size, and an "X" symbol of identical dimensions was overlaid on each image. Five sampling points for melanin assessment were defined: the four endpoints and the center point of the "X". Melanin brightness was measured using Photoshop 2022 (Adobe Inc., USA), and each treatment was performed in triplicate.

Table S1

Table S1. RT-qPCR reaction system for validation of differentially expressed genes

Reaction Component	Volume
TB Green Premix Ex Taq II	10 μL
PCR Forward Primer (10 μM)	0.8 μL
PCR Reverse Primer (10 μM)	0.8 μL
DNA Template (<100 ng)	2 μL
dd H_2O	6.4 μL
Total	20 μL

Table S2

Table S2. Primer of objective gene and internal reference genes

Gene symbol	Forword primer	Reverse primer
EMG1	CGGACACATTCGCTGACTCT	CACTTTTGTGCGAACCCGAG
SGA1	CCATCAAGCACTTTAGCGCC	TCCATCCGGTCCAGGTAGTT
FBP	CATTGTCGGCGATGACGTTC	CGCCCTGGTAATAGTCGGTC
kduD	GTCGCAAGGCCTGGATCTAC	GGAACTCTTCGCAGGGATGT
TYR	TGGATGAAGAAGTTGGCGCT	TGGCCTCTGATTCACGCTTT
ARD1	CCAAGTTCCCCCTCATTCCC	CATGGGCGTTGAACTATGCG
EGLC	AACCAGAAGGACAACGGCAT	CCAGCCAGTCTCGGTAATCC

Text S2. Effects of different concentrations of Hg and Se on pigment production in *O. sinensis*

As shown in Supplementary Fig. S1, melanin accumulation in the liquid culture medium exhibited marked differences among treatments with varying concentrations of Hg and Se. With increasing Se concentration, melanin content in the mycelium showed a continuous accumulation trend, with pigment brightness ranging from 43.9% to 60.3%. Under Hg stress, at a concentration of 2 mg/L, eumelanin accumulation intensified and the medium appeared dark brown (pigment brightness: 7.9%). At 30 mg/L Hg (pigment

brightness: 51.4%), the color was similar to that of the inoculated control group without Hg or Se supplementation (CK3, pigment brightness: 47.2%). At 60 mg/L Hg (pigment brightness: 67%), the color was comparable to that of the uninoculated liquid medium (CK2, pigment brightness: 61.3%). With increasing Hg concentration, melanin production in TZ8-1 mycelium initially increased to a maximum and then declined, with pigment brightness ranging from 7.9% to 67%. At an Hg concentration of 60 mg/L (pigment brightness: 67%, similar to that of the uninoculated medium CK2 at 61.3%), eumelanin production nearly disappeared.

Figure S2

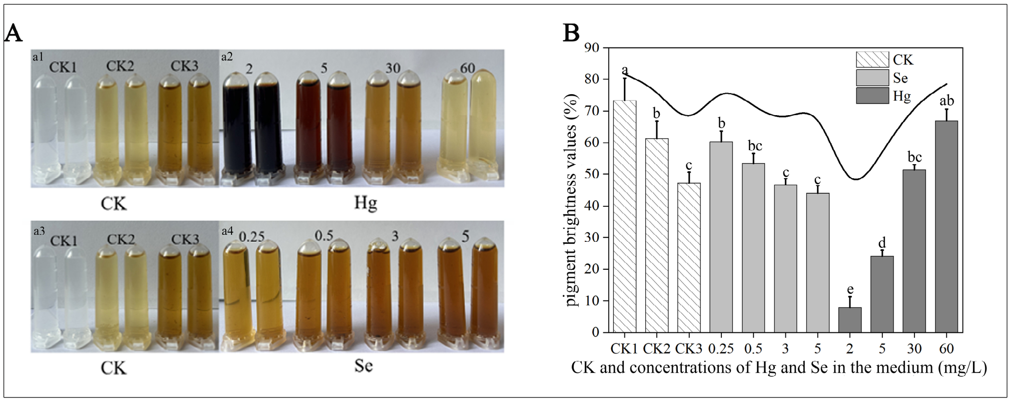

Figure S2. Changes in melanin brightness of *O. sinensis* under different treatments. (A) Visual comparison of culture medium color under different treatments. a1,a3: CK1: sterilized deionized water; CK2: uninoculated liquid culture medium; CK3: inoculated group without Hg and Se supplementation; a2: Hg treatment groups at concentrations of 2, 5, 30, and 50 mg/L; a3: Se treatment groups at concentrations of 0.25, 0.5, 3, and 5 mg/L. (**B**) Statistical graph of pigment brightness under different treatments; Different lowercase letters indicate significant differences among treatments at the P < 0.05 level, and the same letter represents no significant difference

Figure S3

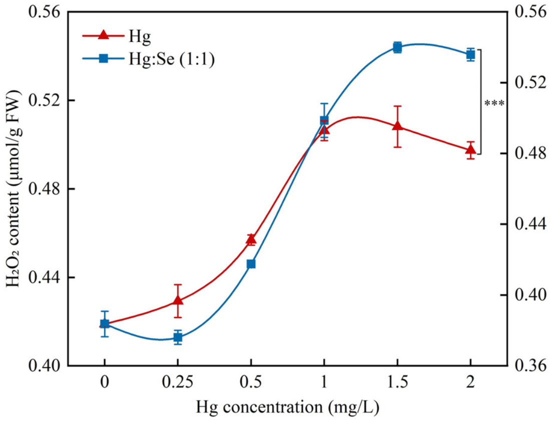

Figure S3. Changes in H_2O_2 content in *O. sinensis* under Hg and Hg+Se treatments. (Asterisks indicate significant differences between groups (*P < 0.05, **P < 0.01, ***P < 0.001)

Table S3

Table S3. Preprocessing results of sequencing data quality

Sample	Raw reads	Raw bases	Clean reads	Clean bases	Error rate(%)	Q20 (%)	Q30 (%)	GC content (%)
CK_1	42.82M	6.47G	42.27M	6.31 G	0.0127	98.32	94.9	59.04
CK_2	43.02M	6.5G	42.44M	6.31 G	0.0128	98.25	94.72	58.88
CK_3	42.91M	6.48G	42.32M	6.26 G	0.0127	98.28	94.77	59.06
Hg_1	43.02M	6.5G	42.41M	6.32 G	0.0129	98.2	94.55	58.87
Hg_2	43.81M	6.62G	43.27M	6.46 G	0.0127	98.32	94.89	58.85
Hg_3	40.58M	6.13G	40.10M	5.97 G	0.0127	98.27	94.74	58.72
Se_1	40.23M	6.07G	39.72M	5.91 G	0.0128	98.23	94.6	58.74
Se_2	42.78M	6.46G	42.23M	6.28 G	0.0127	98.28	94.75	58.7
Se_3	42.89M	6.47G	42.38M	6.31 G	0.0126	98.3	94.8	58.81
Hg+Se_1	46.08M	6.96G	45.48M	6.77 G	0.0128	98.23	94.6	59.19
Hg+Se_2	40.09M	6.05G	39.58M	5.90 G	0.0128	98.23	94.59	59.05
Hg+Se_3	42.76M	6.46G	42.26M	6.31 G	0.0127	98.29	94.76	59.04

Note: (1) Raw reads: total number of raw sequencing reads; (2) Raw bases: total amount of raw sequencing data; (3) Clean reads: total number of reads after quality filtering; (4) Clean bases: total amount of clean sequencing data; (5) Error rate (%): average base error rate of clean data, generally below 0.1%; (6) Q20 (%) and Q30 (%): quality assessment of clean data, representing the percentage of bases with sequencing quality above 99% (Q20) and 99.9% (Q30) relative to total bases, generally Q20 > 85% and Q30 > 80%; (7) GC content (%): percentage of guanine (G) and cytosine (C) bases relative to total bases in the clean data;

(8) Sequencing Depth: total number of reads obtained per sample (basic differential expression analysis: 5–25 million reads; routine transcriptome studies: 20–60 million reads). In general, the Raw/Clean reads data volume is suggested to be > 6 Gb, but this is not an absolute criterion and should be adjusted according to the genome size.

Table S4

Table S4. Fold change and $\log_2$ (fold change) for DEGs in Hg vs CK, Hg+Se vs CK, Hg+Se vs Hg, Se vs CK, and Hg+Se vs Se comparisons

Gene Name	Comparison	Fold Change (FC)	$\log_2$(FC)
EMG1	Hg vs CK	0.4490	-1.1553
EMG1	Hg+Se vs CK	0.4859	-1.0414
EMG1	Hg+Se vs Hg	0.4584	-1.1253
EMG1	Se vs CK	0.4963	-1.0107
EMG1	Hg+Se vs Se	0.4653	-1.1034
FBP	Hg vs CK	0.4774	-1.0668
FBP	Hg+Se vs CK	0.3275	-1.6107
FBP	Hg+Se vs Hg	0.1942	-2.3645
FBP	Se vs CK	0.2257	-2.1473
FBP	Hg+Se vs Se	0.1997	-2.3236
kdhD	Hg vs CK	2.1622	1.1125
kdhD	Hg+Se vs CK	2.1041	1.0732
kdhD	Hg+Se vs Hg	2.3238	1.2165
kdhD	Se vs CK	2.4479	1.2912
kdhD	Hg+Se vs Se	2.0456	1.0325
TYRS	Hg vs CK	2.2052	1.1409
TYRS	Hg+Se vs CK	28.0417	4.8095
TYRS	Hg+Se vs Hg	26.0725	4.7045
TYRS	Se vs CK	13.4705	3.7518
TYRS	Hg+Se vs Se	10.2731	3.3607
ARD1	Hg vs CK	7.6784	2.9408
ARD1	Hg+Se vs CK	19.0291	4.2501
ARD1	Hg+Se vs Hg	2.4943	1.3186
ARD1	Se vs CK	3.1113	1.6371
ARD1	Hg+Se vs Se	4.2935	2.1024
EGLc	Hg vs CK	5.1397	2.3617
EGLc	Hg+Se vs CK	596.8413	9.2212
EGLc	Hg+Se vs Hg	117.4220	6.8756
EGLc	Se vs CK	25.5187	4.6734
EGLc	Hg+Se vs Se	4.0989	2.0352

Note: DEGs identified by DESeq2 analysis ($|\log_2 FC| \geq 1$, p.adjust < 0.05)

Figure S4

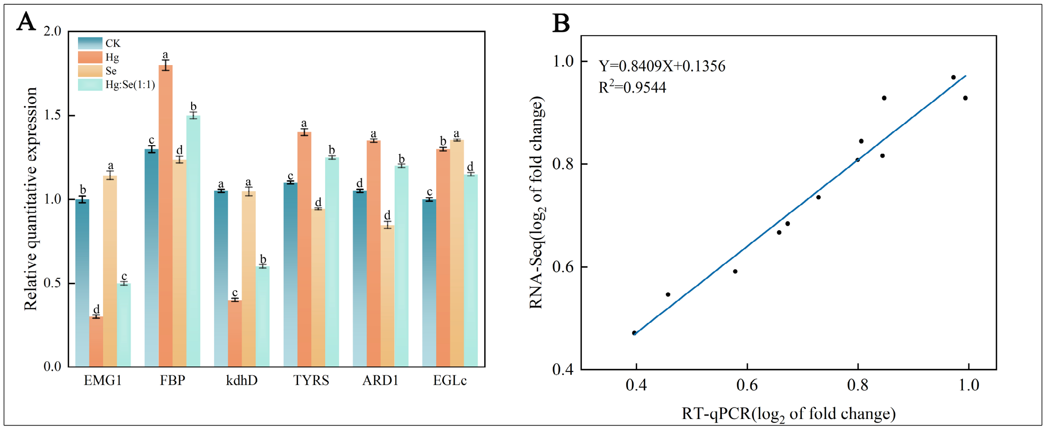

Figure S4. Gene expression levels of *O. sinensis* by RT-qPCR and linear correlation between RNA-seq and RT-qPCR detection results. (**A**) Relative gene expression levels in CK, Se, Hg, and Hg:Se = 1:1 treatments detected by RT-qPCR. (**B**) Linear correlation between $\log_2$ (fold change) values obtained from RT-qPCR and RNA-seq for six key genes. Different lowercase letters indicate significant differences among treatments at the $P < 0.05$ level, and the same letter represents no significant difference.

Figure S5

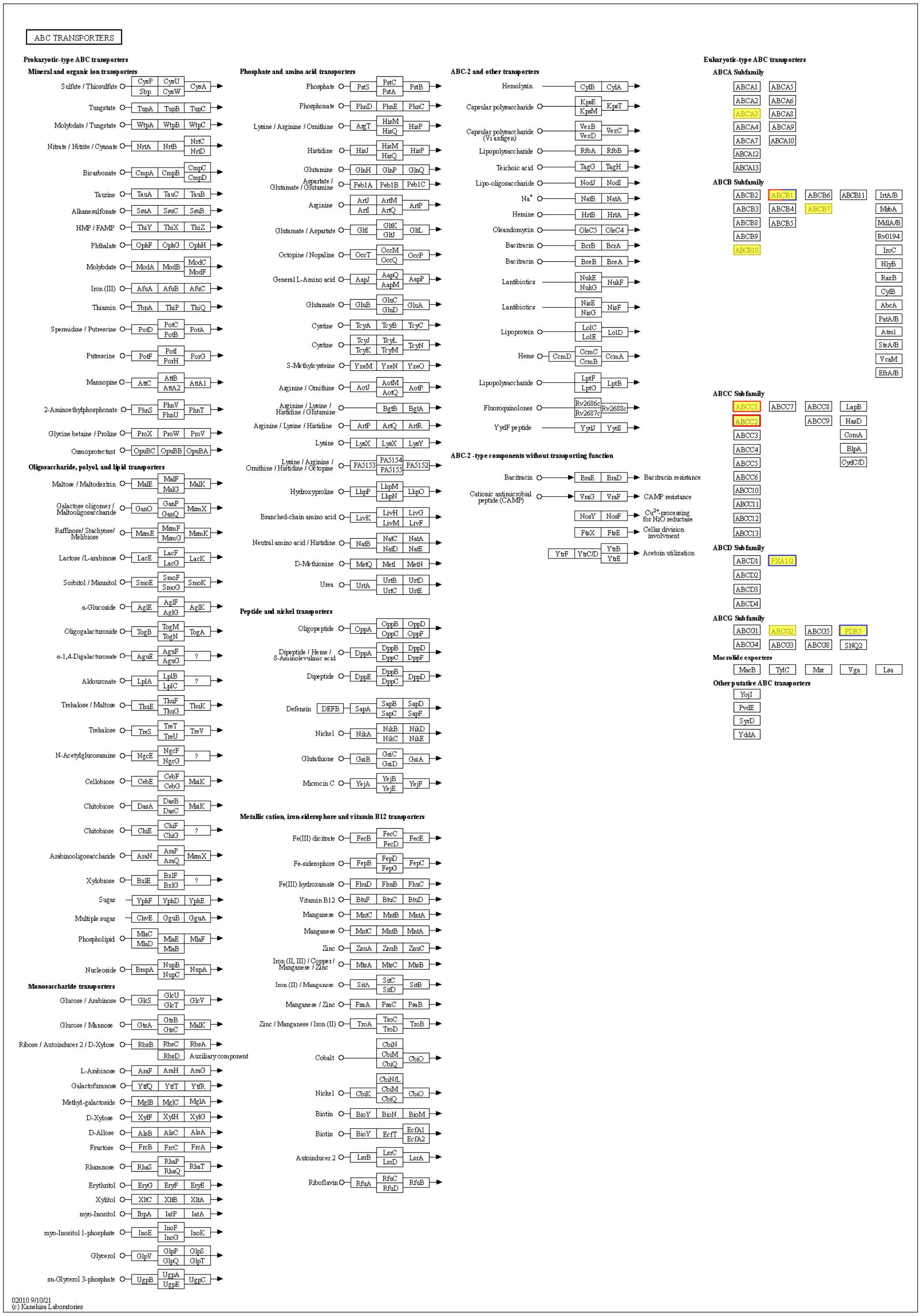

Figure S5. Analysis of the ABC transporter metabolic pathway in *O. sinensis* under Hg+Se treatment compared to Hg treatment alone.
